# Lexical and grammatical arity-reduction: The case of reciprocity in Romance languages

**DOI:** 10.1007/s11049-025-09681-5

**Published:** 2025-09-04

**Authors:** Giada Palmieri, Renato Basso, Júlia Nieto i Bou, Yoad Winter, Joost Zwarts

**Affiliations:** 1 Department of Interpretation and Translation, University of Bologna, Corso della Repubblica 136, Forlì, 47121 Italy; 2 Department of Letters, Federal University of São Carlos, Washington Luís Highway 235 km, São Carlos, SP-310 São Paulo Brazil; 3 Department of Languages, Literature and Communication, Utrecht University, Trans 10, Utrecht, 3512 JK The Netherlands

**Keywords:** Reciprocity, Reflexivity, Romance, Arity-reduction, Lexicon

## Abstract

In many languages, reciprocal meanings are expressed either by grammatical means or by using lexical predicates. The grammatical strategy is productive and may involve derivational affixes (Swahili *-an*) or pronouns (English *each other*) with transitive forms, whereas lexical reciprocity is expressed by a restricted class of intransitive predicates like English *kiss* or *meet*. The situation is more complex in Romance languages, where reciprocal verbal constructions often require a *se* clitic without a clear separation between transitive and intransitive forms. Addressing this puzzle, we propose that Romance languages involve a grammatical/lexical distinction as in other languages. We show that numerous Romance constructions systematically allow *se* omission with certain reciprocals, exhibiting parallel properties to those of lexical intransitives in other languages. A similar observation is made in relation to the distinction between grammatical reflexivity (e.g., English *oneself*) and lexical reflexives (*wash, shave*). Furthermore, we show that the *se* requirement may also be relaxed with transitive verbs, when reciprocity or reflexivity is conveyed by an overt reciprocal/reflexive item (e.g., Spanish *mutuamente* ‘mutually’). The emerging theoretical picture supports an analysis of *se* as a head projection that licenses arity-reduction, though language-specific conditions allow *se* omission when arity reduction is achieved by a lexical reciprocal item or by another overt reciprocal element.

## Introduction

The way in which languages express reciprocal meanings received attention both in theoretical linguistics and typological studies (Frajzyngier and Walker 2000; Nedjalkov et al. 2007; König and Gast 2008; Evans 2011). Two reciprocal strategies have been identified cross-linguistically: lexical reciprocity and grammatical reciprocity. Lexical reciprocity is expressed by a restricted class of intransitive predicates without pronominal elements or other productive derivational strategies. In English, lexical reciprocity is often realized with zero morphology (1). This kind of reciprocity is restricted to verb meanings in the conceptual domain of ‘natural reciprocals’ (Kemmer 1993) and is not possible with just any transitive verb (2).^1^

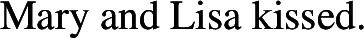

(2)

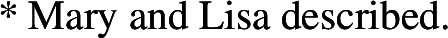

Grammatical reciprocity is the productive strategy by which pronouns, adverbs or affixes lead to reciprocal interpretations with all transitive verbs. In English, grammatical reciprocity requires the elements *each other* or *one another* (Dalrymple et al. 1998) as in (3) and (4).

(3) Mary and Lisa kissed each other.

(4) Mary and Lisa described each other.

Lexical and grammatical reciprocals lead to different interpretations (Kemmer 1993; Carlson 1998, *inter alia*): the grammatical reciprocal construction in (3) can be interpreted with different unidirectional events (for instance, Mary and Lisa kissing each other consecutively on the forehead), whereas its intransitive counterpart in (1) refers to a single collective event (one mutual simultaneous kiss). This semantic contrast reflects a difference in argument structure: grammatical reciprocals are treated as predicates with two arguments bound by a reciprocity operator, and lexical reciprocals as intransitive predicates with one semantically plural argument (Langendoen and Magloire 2003; Dimitriadis 2008a; Winter 2018).

The lexical strategy is overtly manifested in several languages, using morphological forms that characterize reciprocal meanings. For instance, while lexical reciprocals are realized with zero morphology in English and Dutch (Reinhart and Siloni 2005), in Hebrew they are usually realized in the *hitpael* template (Doron 2003), in Modern Greek with non-active morphology (Doron and Rappaport Hovav 2009) and in Hungarian with the verbal marker -*oz* (Rákosi 2008).

In some languages, however, reciprocals do not seem to show any clear distinction between lexical and grammatical processes. This is the case with Romance languages, where the clitic *si*/*se* (in its different realizations, henceforth *se*) is generally required in sentences with transitive verbs that receive a reciprocal interpretation. Such sentences typically receive an additional reflexive interpretation, which is illustrated by the Italian examples in (5).^2^

(5)
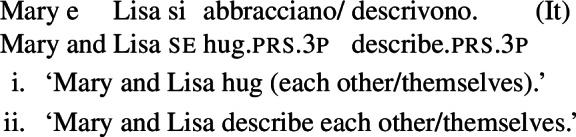

From the surface realization of sentences like (5) we cannot determine whether their interpretation is derived using lexical intransitives such as English *hug*, or using complex transitive constructions with a reciprocal/reflexive operator.

A similar puzzle appears with Romance reflexives. For instance, unlike the English distinction between intransitive *wash* and grammatical reflexives like *wash oneself* or *describe oneself*, Italian generally requires a *se* clitic. Accordingly, the interpretation of plural sentences like (6) is either reflexive or reciprocal, without a clear distinction between lexical and grammatical reflexive usages of *wash*:

(6)

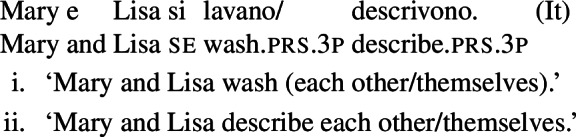

The possible existence of lexical reciprocals and reflexives in Romance has been occasionally mentioned in the literature (Labelle 2008; Doron and Rappaport Hovav 2009; Siloni 2012), but little attention has been dedicated to their characterization. Given the lack of morpho-syntactic cues enabling their immediate identification, this is not a straightforward task. Furthermore, due to the uncertain distinction between grammatical and lexical reciprocal/reflexive processes in Romance, the role of the clitic *se* has been predominantly studied in relation to the grammatical strategy. In this paper we show that the lexical/grammatical distinction systematically appears in Romance, with similar effects to those familiar from other languages. After distinguishing between the lexical and the grammatical reciprocal strategies, we focus on some general aspects of their semantic analysis, and on the contribution of *se* to these strategies. A parallel analysis, with similar motivations, is provided for the lexical/grammatical distinction with Romance reflexives.

When concentrating on the distinction between lexical and grammatical reciprocity/reflexivity, we substantiate the argument in favor of a class of Romance predicates that have a transitive entry and an intransitive reciprocal/reflexive entry, similarly to other languages. Despite the absence of an overt distinction in Italian finite clauses like (5), we find syntactic environments where predicates from a closed class lead to reciprocal/reflexive interpretations all by themselves, without *se* or another overt reciprocal/reflexive element. A language that is especially convenient for illustrating this finding is Brazilian Portuguese (BP). In BP, the clitic *se* is productively associated to reflexivity and reciprocity, similarly to other Romance languages: in (7), *se* is mandatory for the grammaticality of the clause, which gets a reflexive or a reciprocal reading, just like the Italian example in (5). However, there is a handful of verbs that allow a reciprocal interpretation in their bare intransitive form. One example is *abraçar* ‘to hug’ in (8): it receives a reciprocal interpretation either with *se* (8a) or without it (8b).

(7)

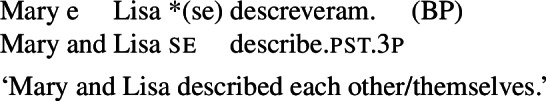

(8)

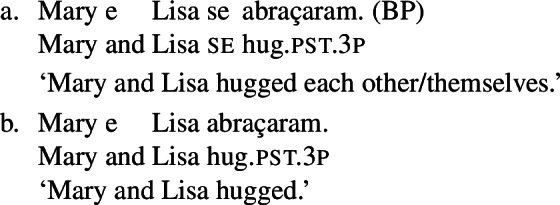

The two configurations with *abraçar* in (8) differ in two respects. First, the reflexivity/reciprocity polysemy only emerges in (8a), whereas its counterpart without *se* in (8b) is unambiguously reciprocal. Second, the sentence in (8b) receives an interpretation that is reminiscent of the ‘single-event’ interpretation of English lexical reciprocals as in (1): (8b) describes a situation where Mary and Lisa are involved in a mutual hug, and it would not be true in a scenario where each of the girls hugged the other in a different moment (e.g., while she was asleep).

We propose that reciprocal interpretations without *se* as in (8b) are due to the lexical meaning of the intransitive verb stem (‘hug’), and that predicates similar to *abraçar* exist in all Romance languages. We focus primarily on data from BP, Catalan, Italian, and Spanish, showing that the possibility of BP to express reciprocity without *se* in (8b) is representative of a broader pattern in Romance. In all four Romance languages, we present constructions where verbs with similar meanings to English intransitives like *hug* lead to reciprocal interpretations without *se*. The peculiarity of BP within the Romance family is that *se* may be omitted in finite clauses, whereas in the majority of Romance languages this only happens in some non-finite constructions. We argue that this syntactic variation does not affect the semantic characterization of lexical reciprocal predicates, which is uniform across the Romance languages we studied. Whenever a Romance verb manifests a reciprocal meaning in some construction without *se* and without any overt reciprocal element, we show that the verb exhibits the semantic characteristics of reciprocal intransitives in English. We reveal a similar pattern in relation to reflexivity: across the four Romance languages that we study, we show that the same constructions that support lexical reciprocity without *se* also support reflexive meanings with verbs that have similar meanings to English lexical reflexives (*shave*, *bathe*).

After presenting evidence for the existence of lexical reciprocity and reflexivity in Romance, we move on to cases of grammatical reciprocity and reflexivity, and to the role of *se* in their derivation. We show that in the syntactic environments where lexical reciprocity and reflexivity emerge without *se*, grammatical reciprocity and reflexivity are also possible without *se* for all transitive verbs, provided that they appear with an overt reciprocal or reflexive operator. For instance, we saw in (7) that BP *descrever* ‘to describe’ cannot lead to a reciprocal interpretation with zero morphology. Yet, with the element *um o outro* ‘one another’, this verb does allow *se* omission, as in the following sentence:

(9)

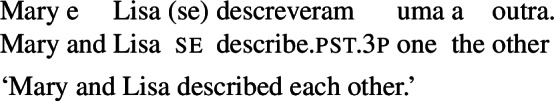

Taken together, these facts lead us to propose that *se* itself is not an exclusive source of reciprocity: elements such as BP *um o outro* have the meaning of reciprocal operators, whereas the reciprocal interpretation of lexical verbs such as *abraçar* ‘to hug’ is due to the inherent intransitive meaning of the verb stem. We will show that analyses of *se* as a marker of valence-reducing operations (Reinhart and Siloni 2005) or as a reciprocal anaphor (Doron and Rappaport Hovav 2009) fail to account for the appearance of *se* with predicates that are inherently reciprocal, and to capture instances of grammatical reciprocals without *se*. By contrast, our findings are consistent with Labelle (2008)’s proposal that *se* is a functional head projection, more specifically Voice. However, against Labelle, we argue that *se* never carries itself the meaning of a reciprocal or reflexive operator. We propose that the role of *se* is purely syntactic: it is a Voice head that marks reflexive and reciprocal predicates, by requiring that no external argument is introduced in Voice. Whether *se* is obligatory depends on the syntax of the clause and on the presence of other elements that mark reflexive/reciprocal interpretations. In the absence of a lexical reflexive/reciprocal verb or of an overtly realized reflexive/reciprocal item, *se* is required to satisfy Condition B, according to which a reflexive predicate must be reflexive-marked (Reinhart and Reuland 1993). Conversely, in the presence of a lexical reflexive/reciprocal verb or of an overtly realized reflexive/reciprocal item, only the syntactic requirements of the construction determine whether *se* is obligatory (e.g., in Italian finite clauses), optional (BP finite clauses) or disallowed (Italian causative clauses).

The paper is structured as follows. In §2 we provide an overview of previous work on lexical reciprocity in Romance languages. In §3 we describe the distribution of *se* across different constructions in four Romance languages, and we identify a group of Romance predicates that may receive a reciprocal interpretation without *se*. In §4 we show that Romance predicates that express reciprocity without *se* have semantic properties that are cross-linguistically associated with lexical reciprocals: reciprocal nominalization (§4.1), semantic drift (§4.2), pseudo-reciprocal interpretation (§4.3), discontinuous reciprocity (§4.4) and acceptability of singular group NPs (§4.5). In §5 we point out the parallelism with lexical reflexivity, considering predicates that lead to reflexive interpretations without *se*. In §6 we explore instances of grammatical reciprocity/reflexivity without *se*. Section §7 presents our analysis of *se* as a functional head projection, which accounts for its distribution and contribution to arity-reduction. In section §8 we provide general conclusions.

## Terminology and previous studies

The term ‘naturally reciprocal’ has been used in the typological literature since Lichtenberk (1985) and Kemmer (1993) to refer to predicates that typically denote reciprocal configurations, and that are often realized with morphological markers associated with the middle voice. In Haspelmath (2007) and Knjazev (2007), the additional term ‘lexically reciprocal’ is employed to refer to the sub-group of the ‘naturally reciprocal’ predicates that express reciprocity without any overt marking (e.g., *fight* or *quarrel* in English). A more encompassing definition of ‘lexical reciprocals’ is provided by Nedjalkov (2007), who defines them as verbs “whose meanings is not a mere sum of the meaning of the base and the meaning of ‘each other’’’ (p.14).

Following Nedjalkov’s definition of ‘lexical reciprocals’, in this paper we use this term to refer to predicates whose reciprocal interpretation does not arise from a productive morpho-syntactic operation, but from an inherent collective meaning of the verb’s intransitive entry. Thus, predicates like *kiss* are assumed to have two distinct entries: a transitive entry (10a) and an intransitive, lexical reciprocal, alternate (10b).

(10)
  - Mary kissed Lisa.
  - Mary and Lisa kissed.

Although many lexical reciprocals have a transitive alternate, some of them do not have a corresponding transitive form. For example, the intransitive verb *talk* does not have a transitive alternate, but an alternate that takes a prepositional complement (11a). The meaning relation between the two *talk* alternates is parallel to the *kiss* alternation in (10). Accordingly, we characterize the collective use of intransitive *talk* in (11b) as a lexical reciprocal.

(11)
  - Mary talked to Lisa.
  - Mary and Lisa talked.

We oppose this notion of ‘lexical reciprocity’ to ‘grammatical reciprocity’: a process whereby reciprocity is derived through a productive strategy, as with *each other* in English. Unlike lexical reciprocity, grammatical reciprocity in English is possible with all transitive verbs and verbs with prepositional complements, whether they have an intransitive reciprocal alternate (12a,12b) or not (12c).

(12)
  - Mary and Lisa kissed (each other).
  - Mary and Lisa talked (to each other).
  - Mary and Lisa described *(each other).

When contrasting lexical reciprocity with grammatical reciprocity, we rely on the theoretical assumption that reciprocal meanings of verbs are lexically associated with the intransitive entry, without any necessary morpho-syntactic process. This assumption leaves room for the possibility that certain languages possess lexical reciprocals that are, on the surface, less distinctly differentiated from grammatical reciprocals than in English. Specifically, in Romance languages there is no clear morpho-syntactic marking that is reserved to lexical reciprocals: verbs with a transitive alternate usually require the element *se* in order to get a reciprocal (or reflexive) meaning. This clitic is not restricted to reflexivity and reciprocity, and it is also used to convey other typical functions of middle forms, including unaccusative, impersonal, passive, and subject-experiencer configurations (Cinque 1988; Chierchia 1995; Dobrovie-Sorin 1998; Rivero 2001; d’Alessandro 2008; Dobrovie-Sorin 2017). The role of the *se* clitic in Romance has been extensively studied in works investigating valence-reducing operations (Grimshaw 1980; Everaert 1986; Reinhart and Reuland 1993; Baauw and Delfitto 2005; Reinhart and Siloni 2005; Doron and Rappaport Hovav 2009; Labelle 2008; Labelle and Doron 2010, *inter alia*). However, the identification and characterization of lexical reciprocal predicates in Romance has not received much attention. This class of verbs has only been occasionally taken into account in theories of Romance reflexives and reciprocals (generally focusing on the former), and it is often treated as an orthogonal question to the grammatical realization of valence-reducing operations.

Reinhart and Siloni (2005) propose a ‘lexicon-syntax’ parameter, by which arity-reducing operations in any language may apply in the lexicon or in the syntax. Thus, in Reinhart and Siloni’s approach, the distinction between lexical and grammatical reciprocity/reflexivity plays a cross-linguistic role, but they do not elaborate on lexical/grammatical distinctions within one and the same language. Reflexivization is claimed to take place through the *bundling* operation, that maps an internal *θ*-role onto the external argument to form a complex *θ*-role. This operation of arity-reduction is illustrated in (13): reflexivization bundling turns a two-place predicate (with two *θ*-roles) into a one-place predicate (with one complex *θ*-role).

(13)
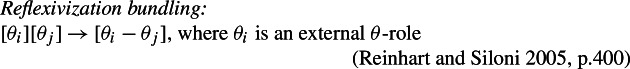

In ‘lexicon languages’, such as English, Dutch or Hebrew, reflexivization and reciprocalization are not productive operations, and lexical reflexive and reciprocal predicates are distinguishable from their counterparts with anaphors. Reciprocal predicates formed in the lexicon are characterized by the absence of ambiguity with reflexive interpretations (Reinhart and Siloni 2005), by a ‘single-event’ interpretation, and by the availability of the discontinuous reciprocal construction (Siloni 2012).^3^ ‘Lexicon languages’ are opposed to ‘syntax languages’, where the reflexive/reciprocal strategy is productive and is assumed to take place in the syntax. This is for instance the case in the Romance family: here, Reinhart and Siloni (2005) propose that the clitic *se* is functional for the bundling operation. This element is therefore assumed to operate on the argument structure, and to be insensitive to the semantics of the verb. Within this account it is observed that there may be instances of lexical reciprocals in syntax languages: Siloni (2012) notes that the French verb *se battre* ‘to quarrel’ displays syntactic and semantic characteristics typical of lexical reciprocals (such as the availability of the discontinuous construction and a ‘single-event’ interpretation). However, no special treatment is reserved to the role of *se* with respect to the reciprocalization of such predicates: *se* is uniformly analyzed as a marker of valence-reduction, regardless of the verbs it combines with. This leaves a gap in the theory: without more semantic content, this does not account for the differences between verbs like *se battre* and other reciprocal verbs that are formed in the syntax.

Labelle (2008) proposes an advancement of the bundling theory, providing a unified analysis of *se* that accounts for cases where this element is responsible for reciprocal interpretations, as well as for cases where reciprocal readings originate elsewhere. Labelle (2008) observes that the French *se* obligatorily appears with verbs that express reciprocity or reflexivity on their own, such as predicates prefixed with *entre-* or *auto-*, respectively. Labelle assumes that *entre-* and *auto-* bind the internal and external arguments, yielding a verb entry with a reciprocal/reflexive interpretation. For instance, *entreregarder* is considered to already denote a mutual configuration, but it nonetheless requires *se* (14).

(14)

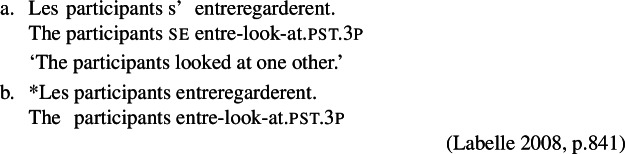

The possibility of *se* to appear with reciprocal verbs rules out a treatment of this element as an arity-reducing morphological unit. However, *se* also appears with simple transitive verbs, and in these cases it is considered responsible for the reciprocal interpretations. To address this distribution, Labelle (2008) treats the French *se* as a functional head projection that introduces the external argument *x* through the agent role (following Kratzer 1996) and identifies it with the object argument of the predicate P (15).

(15)
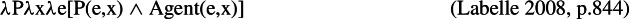

This treatment is meant to unify cases where *se* is the source of reciprocity and cases where it is semantically redundant. With transitive verbs, *se* is needed to express reciprocal interpretations, co-referencing external and internal thematic roles. With predicates prefixed by *entre*-, where the lexical semantics of the verb already has an agent variable (introduced by the prefix), it is assumed that the external argument introduced by *se* is identified with the external argument provided by the verb’s entry. In such cases, *se* does not contribute to the reciprocal interpretation of the constructions, but it is considered obligatory to ensure a coherent interpretation.^4^ While Labelle (2008) recognizes the possibility of *se* to combine with predicates that are already reciprocal, this observation only relies on predicates bearing the productive prefix *entre-*; lexical reciprocal verbs are not considered, and no diagnostics for their identification is provided. One shortcoming of the reliance on grammatical reciprocity is that the analysis is based on the assumption that constructions with *se* are semantically transitive: there are always two distinct thematic roles, that are bound at some stage in the derivation (either by prefixation or by *se*). Semantically, this view is suitable for grammatical reciprocity, but it is in conflict with more recent observations on the meaning of lexical reciprocals, according to which the ‘single-event’ reading must originate from an intransitive entry (Dimitriadis 2008b; Siloni 2012; Winter 2018).

In Doron and Rappaport Hovav (2009), the distinction between lexical and grammatical reflexive/reciprocal entries is taken as a starting point for the development of a twofold account of the Romance *se*. Doron and Rappaport Hovav take the reflexive French *se* as a case study, and propose a syncretism of this element between reflexive morphology and reflexive anaphor. They provide morpho-syntactic arguments for treating the cases in which *se* combines with transitive predicates as instances of anaphoric binding. However, an analysis as a marker of argument identification is reserved to *se* when it is associated with lexical reflexive or reciprocal predicates. Such verbs are identified based on the fact that nominals derived from lexical reciprocals have an inherently collective meaning (16) and on the possibility to receive a reciprocal interpretation without *se* in causative constructions (17).

(16)
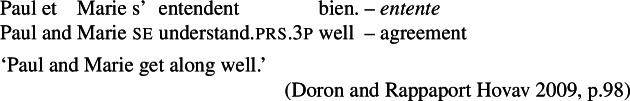

(17)
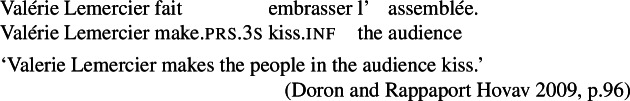

However, as we will show in this paper, various syntactic constructions in Romance allow grammatical reciprocity without *se* under certain circumstances. This fact challenges Doron and Rappaport Hovav’s twofold account of *se*, and will constitute a major element in the evidence that leads us towards an alternative, unified, approach to *se*.

We conclude that despite the overall agreement on the existence of a class of lexical reciprocals in Romance, there is currently no consensus on general tests for identifying those Romance predicates that have a lexical reciprocal (or reflexive) entry, nor on the theoretical implications of the existence of this class. The works outlined above propose different analyses of *se* and of its interaction with lexical reciprocity. However, they all agree on the idea that *se* is semantically responsible for deriving grammatical reciprocal strategies when it combines with verbs that have no reciprocal meaning of their own. In contrast with these previous studies, we will propose a unified treatment of *se*, where its role is purely syntactic as a marker of arity-reduction, and does not derive reflexive or reciprocal interpretations all by itself.

## Lexical reciprocity without *se*

Across languages of the Romance family we can find predicates that express reciprocity without *se*; see Godoy (2008) for BP and Vázquez and Fernández-Montraveta (2016) for Spanish. The Italian predicate *chiacchierare* ‘to chat’ in (18a) receives a collective interpretation in its bare intransitive entry, and cannot combine with the clitic *se*. This configuration is restricted to verbs that do not have a transitive alternate: *chiacchierare* ‘to chat’ cannot take a direct object (18b).^5^

(18)

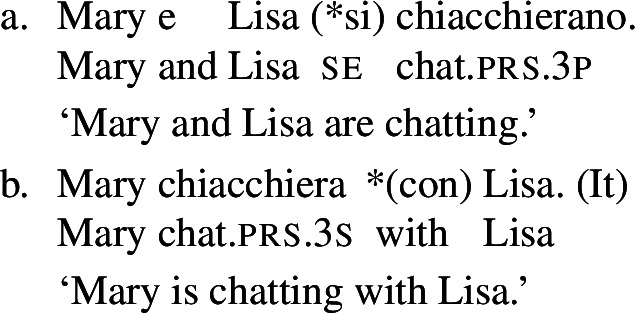

In the absence of any reciprocal marking, the reciprocal interpretation of (18a) must originate from the verb’s entry. Thus, verbs like Italian *chiacchierare* ‘to chat’ fit our definition of lexical reciprocals: they must be stored in the lexicon with an inherent reciprocal meaning. These verbs also fit within the proposed universal that all languages have predicates that denote mutual configurations by themselves (Haspelmath 2007).

However, it is unclear whether the categorization of lexical reciprocals in Romance may also be extended to predicates with a transitive alternate. In many cases, as in the Italian examples in (19), verbs with a transitive entry (19a) require *se* in reciprocal sentences (19b), regardless of whether they denote events typically associated to the class of ‘naturally reciprocal’ predicates (‘hug’) or not (‘describe’).

(19)
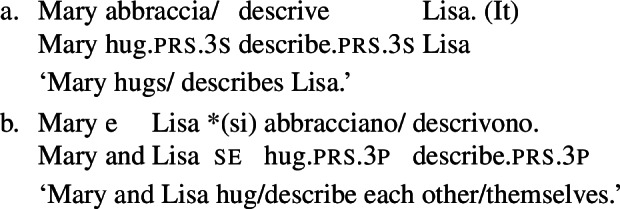

The situation is similar in the vast majority of Romance languages: predicates with a transitive entry consistently require *se* in finite clauses with a reciprocal interpretation. Despite the ubiquity of this phenomenon, the rest of this section will show that all four Romance languages that we studied exhibit constructions where certain verbs with a transitive alternate do express reciprocity without *se*. As we will see, these Romance verbs have meanings that are typical of ‘natural reciprocals’ cross-linguistically, and they give rise to similar semantic effects.

### Finite clauses

In BP finite clauses, most transitive verbs require *se* for obtaining a reciprocal or reflexive interpretation. When *se* appears with a transitive verb and a plural subject, BP sentences uniformly have both reciprocal and reflexive interpretations (20). This is a common situation in other Romance languages as well. However, there is a restricted class of BP transitive verbs where the reciprocal interpretation can also emerge without *se*. For instance, the verb *abraçar* ‘to hug’ expresses a reciprocal meaning both with *se* (21a) and without *se* (21b).^6^

(20)

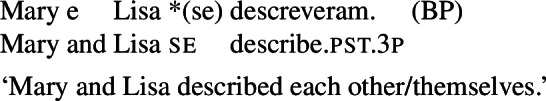

(21)

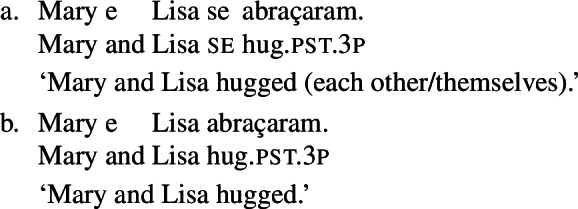

Importantly, the two sentences in (21) differ semantically. The *se*-clause in (21a) displays the common Romance reflexivity/reciprocity ambiguity: it holds true if each individual in the denotation of the subject hugged the other or hugged herself. The meaning of the bare intransitive in (21b) is more specialized: it is only in line with one mutual, collective hug. For instance, unlike (21a), (21b) would not support a scenario with multiple unidirectional hugging events (for instances, where Mary hugs Lisa while Lisa is asleep, and later Lisa hugs Mary while Mary is asleep). Essentially, (21b) only supports a ‘single-event’ interpretation, similarly to its intransitive counterpart in English.

The possible omission of *se* in BP finite clauses has already been observed in the literature (Nunes 1995; Galves 2001; Cyrino 2007; Carvalho 2018), for example with anticausative verbs (22a), or in constructions that receive a medio-passive (22b) or impersonal reading (22c). To the best of our knowledge, however, the optionality of *se* has not been previously recognized with reciprocal or reflexive verbs.

(22)

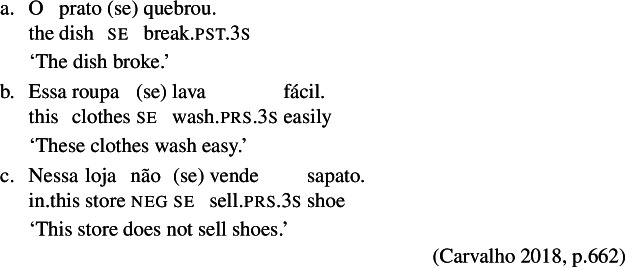

### Analytic causatives

Doron and Rappaport Hovav (2009) notice that in French causative constructions, some verbs with intrinsic reciprocal, reflexive or anticausative interpretations can express these meanings without *se* – although this element would be required in simple finite clauses. This observation holds in other Romance languages too. In Spanish, Catalan, and BP analytic causatives, *se* can be used on the embedded verb to express reflexivity or reciprocity. This process is productive: virtually any transitive verb can be embedded in a causative with *se*, leading to reciprocal or reflexive interpretations. For instance, (23a) holds true if Mary and Lisa described each other or themselves. This is parallel to the situation we have seen in BP finite clauses with *se* in (20). Spanish analytic causatives allow *se* to be omitted, but then the direct object is interpreted as the theme of the action denoted by the embedded verb (Guasti 2006; Folli and Harley 2007). We characterize this as a ‘passive’ interpretation. For example, sentence (23b) means that the subject (“I”) caused Mary and Lisa to be described by an unspecified agent.

(23)

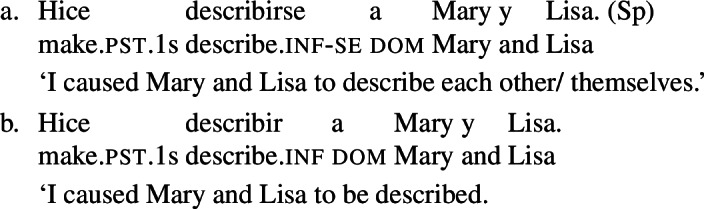

Unlike *describir* in (23b), there are Spanish predicates that allow a reciprocal interpretation in analytic causatives even if *se* is omitted. Let us consider the Spanish verb *abrazar* ‘to hug’. In finite clauses, this verb requires *se* for obtaining reflexive and reciprocal interpretations (24). The verb *abrazar* also gets a reflexive and a reciprocal reading in causatives with *se* (25a). However, unlike *describir* in (23), *abrazar* retains a reciprocal reading in causatives without *se*. Thus, sentence (25b) has a passive interpretation similar to (23b), but it also has a reciprocal reading where the subject (“I”) caused Mary and Lisa to be involved in a mutual hug. Note that in the absence of *se*, no reflexive interpretation emerges in (25b): the sentence is only in line with a passive or a reciprocal interpretation.

(24)

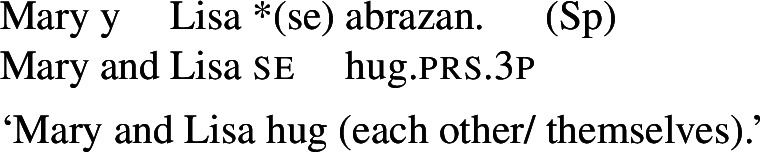

(25)

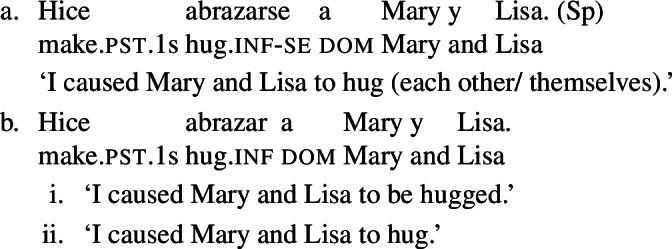

In contrast to Spanish, Italian causatives do not tolerate *se* (Zubizarreta 1985; Guasti 2006). With most transitive verbs in Italian, the only possible interpretation of analytic causatives is passive, similarly to most Spanish transitives in causatives without *se*. For example, sentence (26) is interpreted as claiming that the subject (“I”) caused an unspecified agent to describe Mary and Lisa. By contrast, and similarly to Spanish as well, a restricted set of Italian predicates receive a reciprocal interpretation without *se* in causatives. For instance, with the verb *abbracciare* ‘to hug’, sentence (27) receives a reciprocal interpretation (a mutual hug between Mary and Lisa) on top of the canonical passive interpretation (Mary and Lisa being hugged by an unspecified agent).

(26)

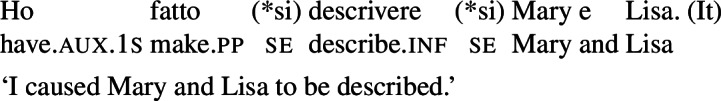

(27)

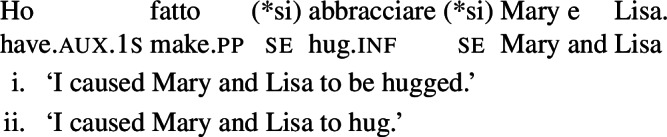

### Absolute constructions

In Spanish and Catalan, another construction reveals the possibility of some verbs to express reciprocity by themselves: the absolute construction with participials, which does not allow *se* in these two languages. When an absolute clause presents a participial followed by an NP, its default interpretation is passive (Hernanz 1991; De Miguel and Lagunilla 2000). For instance, the Catalan example in (28a) states that Teo and Ana left the conference after having been thanked by an unspecified agent. However, with certain verbs, a reciprocal interpretation is available in absolute constructions. Consider for instance the verb *abraçar* ‘to hug’ in (28b), which has an interpretation where Teo and Ana are hugged by a third party, as well as an interpretation where they are involved in a mutual hug.

(28)

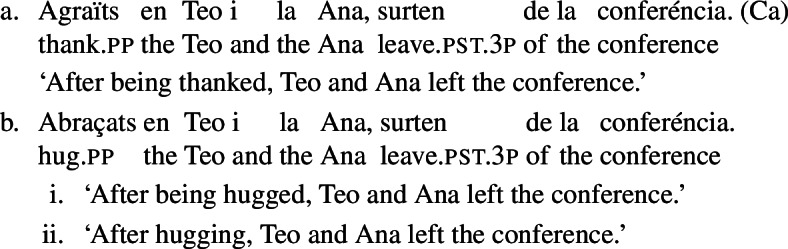

### Overview

Relying on the data presented in this section, we can characterize three groups of predicates in Romance, summarized in Table 1.

**Table 1:** Three classes of Romance verbs

	combines with *se*	reciprocity by itself	example
reciprocal intransitive	–	+	‘chat’
transitive	+	–	‘describe’
reciprocal intransitive / transitive	+	+	‘hug’

In the first class we find verbs like ‘chat’ (18) and ‘discuss’. These verbs do not have a transitive entry, they cannot combine with *se*, and they invariably express reciprocity without any grammatical marking. Similarly to their English counterparts, they get a collective interpretation in their bare intransitive entry.

In the second class there are transitive predicates that combine with *se* and cannot denote reciprocal interpretations without *se* or other additional elements. This is the case of verbs like ‘describe’ or ‘thank’ (20,26,28a). We propose that these verbs are unambiguously transitive, hence they can only express reciprocity through a productive grammatical strategy.

Verbs in the third class have a transitive entry that combines with *se*, but in certain syntactic environments they also get reciprocal readings without *se* or any other additional element. The constructions where omission of *se* is allowed, or even obligatory, differ per language (Table 2). Despite this syntactic variation, the meanings of these verbs are remarkably similar to those of lexical reciprocals in other languages; they all fall into Kemmer (1993)’s categorization of ‘naturally reciprocal’ predicates. Furthermore, in the absence of *se* they all unambiguously lead to the type of ‘single-event’ readings that characterize lexical reciprocals cross-linguistically (§4.3). When reciprocity emerges from the verb alone, the reflexive/reciprocal ambiguity that is typical of Romance *se*-constructions disappears. A summary of the environments where *se* can be omitted with these verbs is given in Table 2.

**Table 2:** Constructions where reciprocity emerges without *se*

	BP	Italian	Spanish	Catalan
finite clauses	+	–	–	–
analytic causatives	+	+	+	+
absolutes with participial	–	–	+	+

Based on these observations, we take reciprocal readings without *se* (and without any other reciprocal marking) to be an indication of lexical reciprocity. We propose that verbs that allow reciprocity with and without *se* have two entries: a transitive entry and an intransitive entry with a lexical reciprocal meaning. A common example of the kind of meaning that is often conveyed by such verbs is ‘hug’; verbs with this meaning have a transitive alternate and can express reciprocity without *se* in all four languages studied here (21b,27,25b,28a). (29) provides a more comprehensive list of English translations of lexical reciprocal verbs with a transitive alternate in these four languages: BP, Catalan (c), Italian (i), and Spanish (s).^7^

(29) **Lexical reciprocals with a transitive alternate:**
  ‘break up’ (c,i,s); ‘confer’ (bp,c,i,s); ‘date’ (bp,i); ‘greet’ (bp,i); ‘hug’ (bp,c,i,s); ‘kiss’ (bp,c,i,s); ‘know each other’ (i); ‘marry’ (bp,c,i,s); ‘meet’ (bp,c,i,s); ‘run into each other’ (c,i,s).

We should stress that under our assumptions, the possibility of a verb to denote reciprocity by itself can be used as a diagnostic for having an intransitive reciprocal entry only under two conditions:

(i) Lexical reciprocals with a transitive alterate do require *se* in many environments other than those in Table 2. For example, the Italian verb *abbracciare* ‘to hug’ expresses reciprocity without *se* in causatives (27), but requires *se* in finite clauses (19b).
(ii) As will be discussed in §6, reciprocal interpretations may emerge in the absence of *se* in constructions as in Table 2, as long as there is an overt reciprocal element. An example are BP finite clauses: if the reciprocal pronoun *um o outro* ‘one another’ is present, *se* can be omitted with all transitive verbs (e.g., *descrever* ‘describe’ in (9)).

With these caveats, we formally define ‘lexical reciprocals’ in Romance as follows:

(30) **Romance lexical reciprocals**: *In a Romance language, we characterize as lexical reciprocals those verbs for which there are syntactic constructions (whose identity is determined by language-specific factors) where a reciprocal interpretation emerges without* se *or another reciprocity element.*

With this notion of lexical reciprocals in Romance, the next section demonstrates that these predicates share semantic properties with lexical reciprocals in other languages.

## Properties of Romance lexical reciprocals

The properties of lexical reciprocal predicates have been explored in many works, typological (Kemmer 1993; Knjazev 2007; Haspelmath 2007), theoretical (Rákosi 2008; Dimitriadis 2008b; Doron and Rappaport Hovav 2009; Siloni 2012; Winter 2018), and experimental (Gleitman et al. 1996; Kruitwagen et al. 2022). In this literature there is an agreement that lexical reciprocals have a different interpretation from their grammatical counterparts, and that they may appear in constructions where grammatical reciprocity is blocked. In this section, we review these properties and show that they consistently appear with the Romance verbs that we characterize as lexical reciprocals. This supports our claim that the ability of a Romance verb to express reciprocity without additional elements reflects the same phenomenon that is cross-linguistically characterized as lexical reciprocity.

### Nominalizations

One property of the Romance predicates that we characterize as lexical reciprocals is the possibility to form nominals with a reciprocal interpretation. Doron and Rappaport Hovav (2009) notice that certain French verbs that they consider lexical reciprocals can be nominalized and keep a reciprocal interpretation (see (17) in §2). This observation can be extended to other Romance languages. Consider for example the Italian verb *incontrare* ‘to meet’, characterized as a lexical reciprocal in (29). The nominal derived from this verb has an inherent reciprocal interpretation: its use in (31) refers to a meeting *between* Mary and Lisa.

(31)
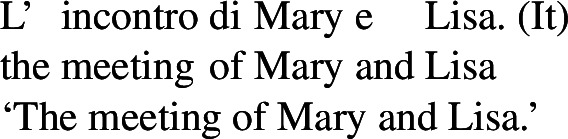

However, verbs often miss nominalized forms. For this reason, nominalizations do not always provide an opportunity to test the verb’s reciprocity. For example, the Italian predicate *lasciare* ‘to leave/break up’ cannot be nominalized, although it can express reciprocity without *se* in causatives, and it has a meaning that is cross-linguistically common among lexical reciprocals. Thus, although we adopt Doron and Rappaport Hovav’s proposal that reciprocal nominalization can *only* appear with lexical reciprocal verbs, we emphasize that this property is not a necessary characteristic of lexical reciprocals under our diagnostics.

### Semantic drift

A common phenomenon among verbs that we categorize as lexical reciprocals is that they do not fully preserve the meaning of their transitive alternate. For example, the Italian verb *trovare* has a transitive entry with the meaning ‘to find’ (32), as well as a logically distinct intransitive meaning: ‘to have a planned meeting’ as in (33). As with all transitive predicates, the ‘find’ meaning of *trovare* can receive a reciprocal interpretation through the grammatical strategy as in (34).^8^

(32)

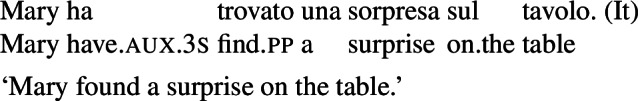

(33)

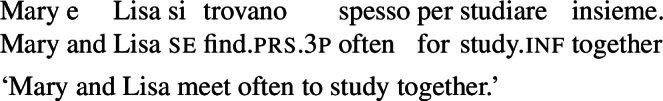

(34)

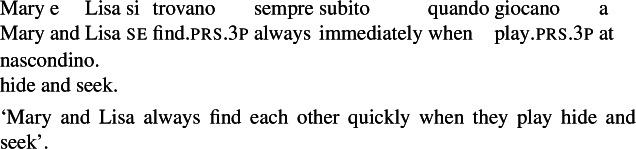

Semantic drift has been observed for verbs with an inherent reciprocal meaning across several languages (Kemmer 1993; Haspelmath 2007; Siloni 2012). This phenomenon results in reciprocal verbs not having a corresponding transitive base; accordingly, such verbs cannot be analyzed as the outcome of a productive strategy where a reciprocity operator applies to a transitive entry. Similarly to nominalizations, we take semantic drift to be an indication of a lexical reciprocal entry, even though we do not expect it to be a necessary characteristic of all verbs of this class.

### Pseudo-reciprocal interpretations

Cross-linguistically, grammatical and lexical reciprocity lead to different interpretations. In events with two participants, grammatical reciprocals describe two different events, where in each event the same binary relation holds between the participants in a different direction. The resulting reciprocity is the accumulation of these different ‘unidirectional events’. By contrast, lexical reciprocals describe a single collective event (Carlson 1998) that typically involves two ‘unidirectional sub-events’. To illustrate this contrast in English, let us consider the grammatical reciprocal form with *each other* in (35a). This sentence is in line with an interpretation that involves different kissing events, where Mary kissed Lisa and Lisa kissed Mary (e.g., on the forehead). The two events do not have to be simultaneous or related to some collective act of Mary and Lisa, but they can. By contrast, the lexical reciprocal form in (35b) does not allow two independent unidirectional kisses and it can only describe a single kissing event between the two people (e.g., a romantic kiss on the lips).

(35)
  - Mary and Lisa kissed each other.
  - Mary and Lisa kissed.

Grammatical reciprocity is central to studies that explore the core meanings of reciprocal elements like *each other*, and their relation with contextual information and predicate concepts (Dalrymple et al. 1998; Beck 2001; Sabato and Winter 2012; Mari 2013; Poortman et al. 2018). We will not delve into all the possible configurations supported by English *each other*, nor into the contrast between “weak” (=partial) and “strong” (=maximal) reciprocity. For the sake of simplicity, we will restrict our attention to reciprocal configurations involving only two entities. For situations with more than two participants, we assume that *se*-less Romance sentences are interpreted like the parallel intransitive sentences in English. The exact interpretation of such sentences in relation to weak or strong reciprocity has not been studied in the literature even with respect to English, hence we have to put it aside.^9^ Our only prediction here is that whatever reciprocity mechanism is uncovered with such reciprocal intransitive sentences in English, it should be uncovered in Romance as well.

Grammatical reciprocal forms with two participants (35a) generally lead to equivalences with a conjunction between two opposite ‘unidirectional’ statements, as in (36).^10^

(36) *x* and *y* kissed each other ⇔ *x* kissed *y* and *y* kissed *x*

By contrast, the interpretation of lexical reciprocal predicates is not exhausted by this equivalence. Winter (2018) illustrates that different lexical reciprocals show different entailments between the collective intransitive form and the two unidirectional statements. Some lexical reciprocals are indeed characterized by a mutual entailment between the collective form and multiple unidirectional relations (37); this equivalence is defined by Winter as *plain reciprocity*. Winter argues that lexical reciprocals that show this equivalence, e.g., ‘meet’, have a symmetric transitive alternate, as illustrated in (38).

(37) *x* and *y* met ⇔ *x* met *y* and *y* met *x*

(38) *x* met *y* ⇔ *y* met *x*

Many lexical reciprocals do not show plain reciprocity and are not symmetric. For instance, the reciprocal entry of the verb *divorce* in (39) does not entail two unidirectional relations: a divorce can be initiated by only one individual.^11^ There are also lexical reciprocals for which the reverse entailment does not hold, i.e., where multiple unidirectional relations do not entail a collective form: in (40) two unidirectional kisses do not imply the occurrence of a mutual kissing event.^12^

(39) *x* and *y* divorced ⇏ *x* divorced *y* and *y* divorced *x*

(40) *x* and *y* kissed ⇍ *x* kissed *y* and *y* kissed *x*

The lack of entailment relations in (39) and (40) is characteristic of lexical reciprocals whose transitive alternate is not symmetric. We use the term *pseudo-reciprocal* to encompass the interpretations that characterize the two kinds of lexical reciprocals: plain reciprocity (37) with its characteristic symmetric equivalence (38) and non-plain reciprocity, where this equivalence fails in one of its two directions (39,40). Pseudo-reciprocity allows us to semantically distinguish lexical reciprocity from grammatical reciprocity. Pseudo-reciprocity primarily emerges with lexical reciprocal verbs (e.g., ‘divorce’, ‘break up’, ‘collide’), and not with grammatical constructions like ‘each other’ pronominals. It should be emphasized that in this paper we are not aiming to give any systematic account of the lexical semantic processes that underly pseudo-reciprocity. Rather, lack of plain reciprocity is simply used as a semantic diagnostic for lexical reciprocity. For more ideas on the semantic principles that underly lexical reciprocity, see Dimitriadis (2008b), Winter (2018), Kruitwagen et al. (2022), among others.

As we will now show, Romance languages show interpretative differences between grammatical and lexical reciprocals parallel to other languages. *Se*-clauses with unambiguously transitive verbs get the same interpretation as English forms with *each other*. For instance, the reciprocal reading of sentence (41) entails that Mary described Lisa and Lisa described Mary.

(41)
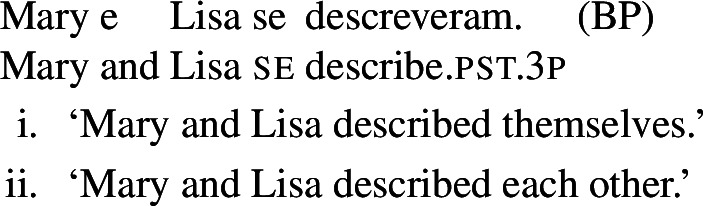

Romance lexical reciprocals get pseudo-reciprocal interpretations. This is most easily observed in constructions without *se* (or other overt reciprocal markers): such cases only show pseudo-reciprocal readings. By contrast, *se*-clauses are consistently in line with the same range of reciprocal interpretations as the corresponding English clauses with *each other*. This contrast can be noted with lexical reciprocals like ‘kiss’ that show a non-plain interpretation.^13^ For instance, the BP example in (42a) and the Spanish example in (43a) have a plain reciprocal interpretation, in line with the equivalence in (36). Both sentences are true if there were at least two unidirectional kisses between the participants. In particular, both sentences are true in a scenario where Mary and Lisa each kissed the other on the forehead in different moments. By contrast, their counterparts without *se* in (42b) and (43b) cannot get an interpretation where each girl was kissed by the other in a different moment: they necessarily denote a mutual kiss. Note that a scenario with a single, mutual kissing event is also supported by (42a) and (43a): the pseudo-reciprocal reading of the lexical reciprocal predicate remains accessible in the presence of *se*.^14^

(42)
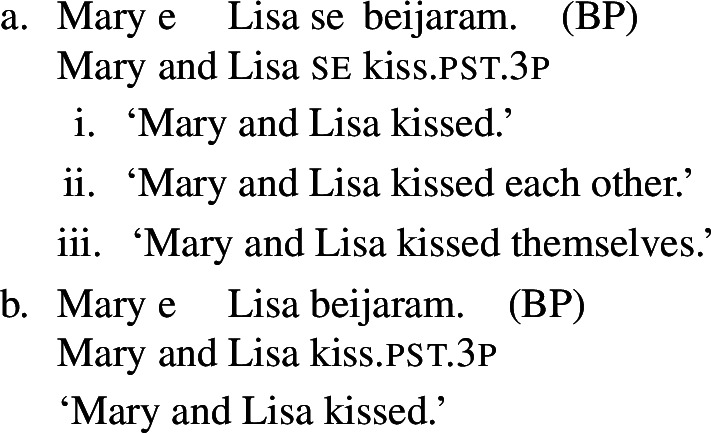

(43)
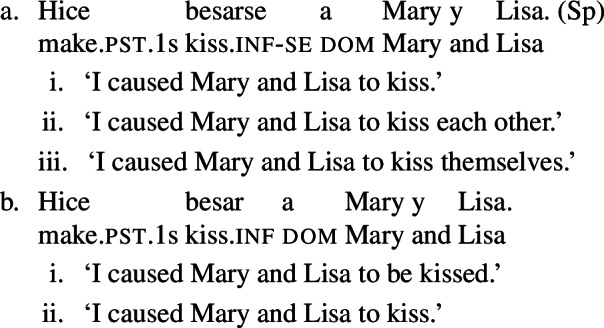

Furthermore, in (42a) and (43a) the presence of *se* correlates with the availability of a reflexive interpretation. A reflexive reading is generally dispreferred with verbs that have a lexical reciprocal entry, but it is not logically excluded. A scenario where Mary and Lisa each kissed herself would be supported in (42a) and (43a), but utterly inaccessible in the absence of *se* (42b,43b).

This evidence illustrates that the only reciprocal interpretation licensed in Romance constructions without *se* (or another reciprocal marker) is the pseudo-reciprocal reading that is associated with lexical reciprocals in other languages. In contrast, *se*-clauses featuring lexical reciprocal predicates are ambiguous between three readings: (i) a pseudo-reciprocal reading, due to lexical reciprocity; (ii) a plain-reciprocal reading, due to grammatical reciprocity; (iii) a grammatical reflexive reading.

Our assumption that *se*-clauses are ambiguous, rather than underspecified, between lexical reciprocal entries and grammatical reciprocal entries is supported by conceptual and empirical considerations. An analysis in terms of underspecification would treat the pseudo-reciprocal and the plain reciprocal readings as two possible senses of the same unambiguous reciprocal construction. This would not explain the different truth conditions of clauses with and without *se* as in (42) and (43). Sentences without *se* like (42b) and (43b) do not support plain reciprocity: they unambiguously require the collective (“reciprocal”) activity of the plural agent to occur in one event. This lack of plain reciprocity is cross-linguistically manifested with intransitives like English *kiss*. When *se* does appear, as in (42a) and (43a), plain reciprocal situations are allowed. This parallels the interpretation of grammatical reciprocals with *each other* in English. Thus, it is natural to explain the licensing of these additional situations by our assumption that adding *se* gives rise to an additional reading of grammatical reciprocity. Our ambiguity proposal is further supported once we look at the zeugma test. Let us consider a scenario where (i) Mary unilaterally hugged Lisa, and Lisa unilaterally hugged Mary in a different moment, (ii) Irene and Bea were involved in a mutual hug, where Irene did not actively wrap her arms around Bea (for instance, because her arms were occupied carrying bags).^15^ In such a scenario, the sentence in (44) below is not easily accepted.^16^

(44)

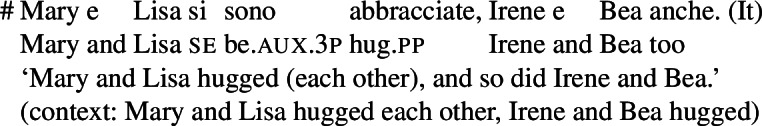

Thus, we argue that pseudo-reciprocal interpretations and plain reciprocal interpretations stem from two distinct entries in the lexicon: an intransitive reciprocal entry, and a transitive entry, respectively. Crucially, we do not argue that *se* itself is ambiguous: in §7, we will provide a unified analysis of this element.

### Discontinuous reciprocal construction

Another property that characterizes Romance lexical reciprocals is the availability of the so-called ‘discontinuous reciprocal construction’, a construction where the logical subject of a reciprocal predicate is split into two parts: one part is encoded as syntactic subject, while the other is in a complement introduced by a comitative preposition (45). It has been noted since Kemmer (1993) that in languages with an overt distinction between lexical and grammatical reciprocity, the discontinuous reciprocal construction is restricted to lexical reciprocals.^17^ In the Greek examples below, discontinuous reciprocity is allowed with the lexical reciprocal ‘kiss’ in (45a), but it is ungrammatical with the productive quantificational strategy in (45b).

(45)

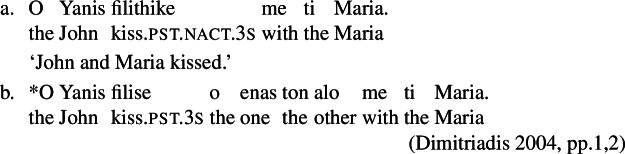

Dealing with Romance languages, Siloni (2012) discusses discontinuous reciprocals in French and Romanian, and proposes that they are restricted to reciprocal verbs formed in the lexicon. For Italian, felicitous instances of the discontinuous reciprocal construction have been noted by Mocciaro (2011), suggesting that they only occur with symmetric verbs like ‘meet’. However, this generalization does not cover all the verbs that show discontinuous reciprocity in Italian. We observe that for many speakers, discontinuous reciprocity is also possible with non-symmetric predicates that belong in the class of lexical reciprocals (46). Discontinuous reciprocity is not unanimously accepted with certain lexical reciprocals, such as *baciare* ‘to kiss’ or *abbracciare* ‘to hug’, and some authors have considered it ungrammatical with these verbs (Dimitriadis 2004; Mocciaro 2011). However, cases where also these verbs appear in the discontinuous construction are accepted in spoken language, and despite their marginality in formal registers, attested examples can be readily found (47). By contrast, the discontinuous reciprocal construction is not felicitous with unambiguously transitive predicates (48).

(46)

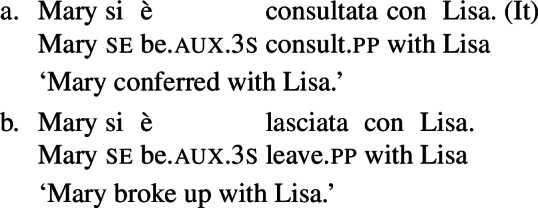

(47)

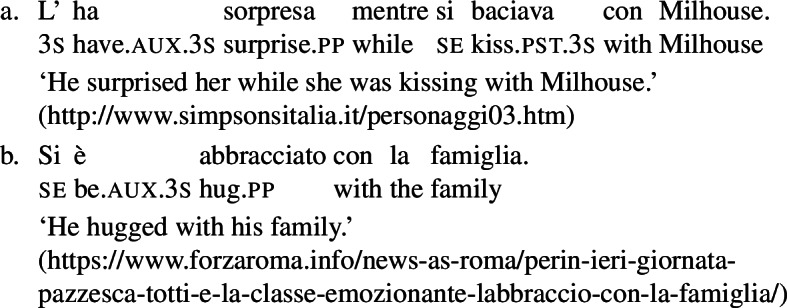

(48)

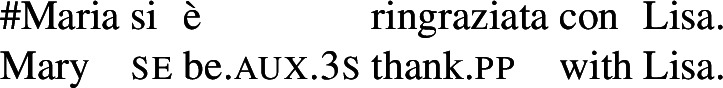

Below we provide more attested examples with lexical reciprocals from Spanish (49), Catalan (50), and BP (51). In these languages too, the discontinuous reciprocal construction is allowed with the verbs that we propose to treat as lexical reciprocals, in line with the cross-linguistic property of this class of verbs. As shown by (51), in BP the omission of *se* is optional in this construction.^18^

(49)

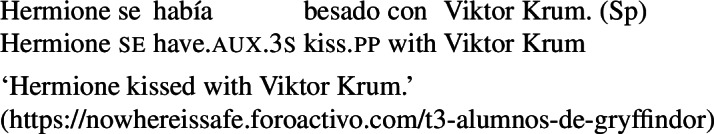

(50)

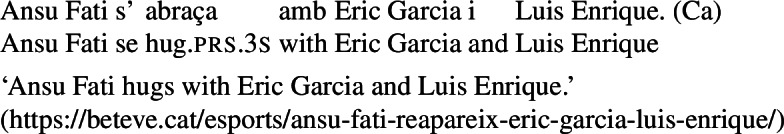

(51)

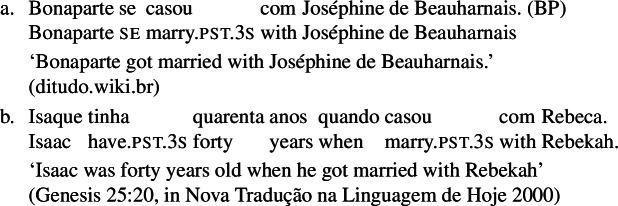

The vast majority of the examined predicates that can express reciprocity without *se* allows discontinuous reciprocity. It is hardly surprising that not all of them are unanimously accepted: this kind of idiosyncrasy is also found in languages with an overt lexical/grammatical reciprocity distinction. In English, for instance, the non-symmetric transitive verb ‘hug’ does not take a reciprocal ‘with’ (52a), whereas ‘fight’ does (52b), although both verbs have intransitive reciprocal entries.

(52)
  *Mary hugged with Lisa.
  - Mary fought with Lisa.

### Singular group NPs

Another characteristic of Romance lexical reciprocals is the possibility of expressing reciprocity with morpho-syntactically singular group NPs. These are NPs headed by singular nouns like *committee*, *team*, and *choir* that refer to collections, usually of animate entities. Barker (1992) defines group nouns in English as those nouns that can take a plural but not a singular *of*-complement, as in (53).

(53) A team of women/*woman.

As noted by Authier and Reed (2018) for French and English, group NPs support some kinds of reciprocal interpretations. In English, morpho-syntactically singular group nouns can act as the subject of lexical reciprocal verbs, allowing an interpretation where the members of the group are mutually involved in the action described by the verb (54a). By contrast, as observed in Barker (1992), English singular group NPs cannot serve as antecedents for *each other*, as *each other* is generally incompatible with singular predication (54b).^19^

(54)

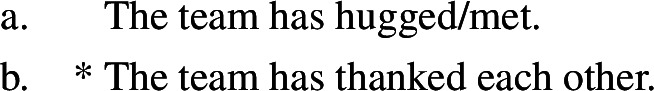

In Romance languages, unambiguously transitive verbs with *se* do not get a reciprocal interpretation with singular group NPs. The BP example in (55) only has a reflexive interpretation, for instance where some representative member(s) of the team described the team as a whole.

(55)
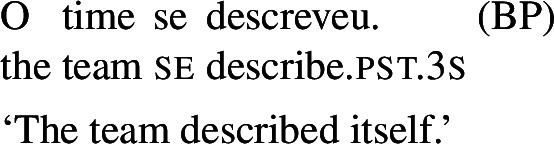

By contrast, the verbs that we characterize as lexical reciprocals can express reciprocity with group NPs and singular agreement. This is shown in the BP example in (56), which is felicitous under the collective reading where the members of the team were involved in a hug. The same holds for Italian (57), Spanish (58), and Catalan (59).

(56)

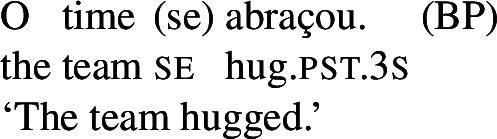

(57)

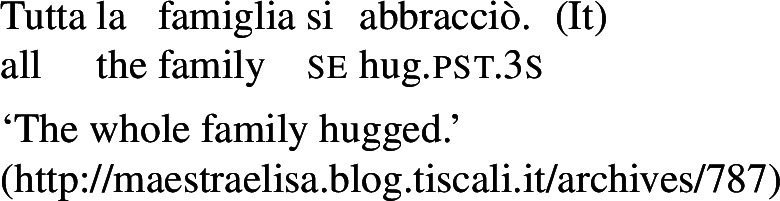

(58)

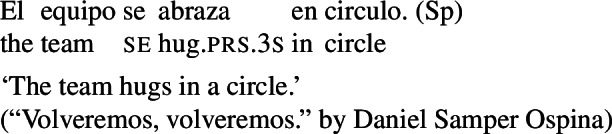

(59)

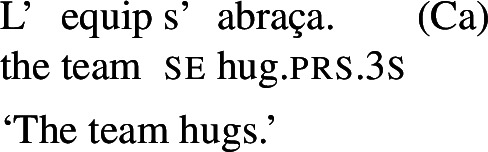

### Romance lexical reciprocals: Summary

In this section we have explored a class of Romance predicates that express reciprocity by themselves, with or without the typical clitic *se*. In a set of language-specific constructions where *se* may or must be omitted, these verbs express reciprocity without any additional reciprocal element, and without the reflexive/reciprocal polysemy that characterizes *se* constructions. We propose that this property indicates a class of *lexical reciprocal verbs*, which other languages exhibit with similar verbal concepts.

In addition to the licensing of *se* omission, we have demonstrated that Romance lexical reciprocals show other properties that are characteristic of lexical reciprocals in other languages. With or without *se*, these verbs consistently support a pseudo-reciprocal interpretation, discontinuous reciprocity using ‘with’, and reciprocal readings with singular group subjects. A comprehensive list of lexical reciprocals we identified in the four Romance languages we study is provided in the Appendices, together with more examples of their properties.

## The case of lexical reflexivity

In this section, we propose that a parallel lexical/grammatical opposition is observed with respect to Romance reflexives. We identify Romance predicates that receive a reflexive interpretation without *se* or another reflexive item, and we argue that they have a lexical reflexive entry. This strengthens our claim that the distinction between lexical and grammatical valence-reducing alternations is general in Romance. The observed facts on reflexivity also support the generalization that the reflexivity/reciprocity polysemy only occurs in the presence of *se*.

### Lexical reflexivity without *se*

We identify lexical reflexive verbs using the same constructions where we observed the emergence of lexical reciprocity without *se*. These constructions vary per language, as previously summarized in Table 2 (§3.4).

In BP, *se* can be omitted in finite clauses. In example (60) below, the BP verb *depilar* ‘remove body hair, shave’^20^ supports situations where Mary shaved herself, similar to the English translation. We propose that this fact categorizes *depilar* as a lexical reflexive in BP.

(60)

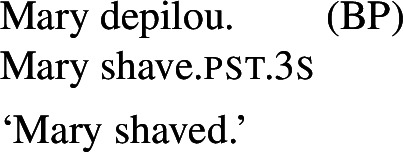

In Catalan, Spanish, and Italian, similar reflexive interpretations emerge in other environments where *se* can (or must) be omitted. Consider for instance the Italian verb *lavare* ‘to wash’ in the analytic causative in (61). Like all Italian causatives with transitive verbs, this sentence has a passive reading, where the subject caused Mary to be washed. Crucially, the verb ‘wash’ in (61) also allows a reflexive reading, where Mary washed herself.

(61)
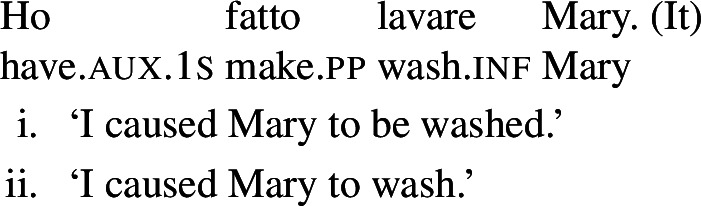

Similar facts hold for analytic causatives in BP, Spanish, and Catalan. Accordingly, we propose that verbs like Italian *lavare* ‘to wash’ have an intransitive reflexive entry similar to English.

Absolute constructions in Catalan and Spanish also support reflexivity without *se*. For instance, the Spanish verb *afeitar* ‘to shave’ in (62) has a reflexive interpretation, on top of the passive interpretation that is standard with transitive verbs in absolute clauses.

(62)
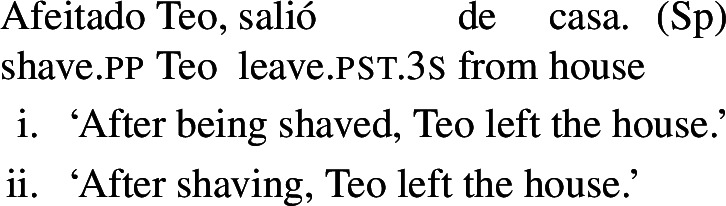

The same holds for absolute clauses in Catalan, where the verb *afaitar* ‘to shave’ (among others) leads to the same array of interpretations as the Spanish version in (62). Accordingly, we propose that verbs like Spanish *afeitar* and Catalan *afaitar* have an intransitive reflexive entry.

### Pseudo-reflexive interpretations

Semantically, there are two critical facts to be observed with respect to the reflexive interpretations above. First, verbs that allow reflexivity without *se* (or another reflexive element) support an interpretation that is subtly but critically different from the meaning generated for transitive verbs with reflexive pronouns. Second, in cases of reflexive interpretations without *se*, no reciprocal meaning emerges.

Considering the first point, let us note a well-known fact: grammatical reflexivity requires identity between two thematic roles. For instance, in (63) the subject is the agent and the patient of the event denoted by the verb: Ali was the entity who shaved/described Ali. It has been noted that instances of grammatical reflexivity are also in line with a so-called ‘proxy’ reading, where the referent of the object is a sufficiently close copy of the referent of the subject, so that it functions as a proxy for it (Jackendoff 1992; Lidz 1997; Reuland 2001). For instance, the English clauses with *himself* in (63) hold true in a scenario where Ali, in the context of a visit to a wax museum, shaved or described a statue of himself. We refer to the interpretation of grammatical reflexives as *plain* reflexivity.

(63)
  - Ali shaved himself.
  - Ali described himself.

Lexical reflexives are semantically distinguished from such cases of grammatical reflexivity (Doron and Rappaport Hovav 2009; Spathas et al. 2015; Haspelmath 2022). First, across languages, speakers accept lexical reflexives in scenarios where the subject is a willing patient (mentally and consensually involved in the event), whereas the agent physically carrying out the action coincides with a different entity. For instance, the English intransitive form in (64) is acceptable if Ali was the one who performed the act, but also if Ali went to the barbershop for a shave. Importantly, the latter possibility is ruled out for the grammatical reflexive in (63a). Moreover, the ‘proxy’ reading is excluded with lexical reflexive entries: unlike (63a), (64) does not support a reading where Ali shaved a statue of himself.

(64) Ali shaved.

We refer to this interpretation of lexical reflexives as *pseudo-reflexive*. We standardly assume that it emerges from the verb’s intransitive entry, and not from any process of argument binding in the syntax.

Grammatical reflexives do not require volition of their agent and patient (which, except in ‘proxy’ readings, are identical).^21^ For example, in an unfriendly scenario where Ali was forced to shave himself against his will, the grammatical reflexive form in (63a) may be considered true. By contrast, volition from the argument may improve the acceptability of lexical reflexive forms, and make them acceptable in situations where the subject does not correspond to the entity physically performing the action. Thus, in case someone shaved Ali against his will, (64) is deviant. We can summarize these semantic differences between grammatical reflexives and lexical reflexives as follows:

*Grammatical reflexives require identity between two arguments of a binary predicate (possibly by proxy), but not all lexical reflexives require strict identity.*

*Lexical reflexives may require more volition of their argument than parallel grammatical reflexives.*

We standardly assume that the identity requirement in grammatical reflexives results from the binding of the reflexive pronominal argument by the other argument. Like in the case of pseudo-reciprocal interpretations, we assume that the reading of lexical reflexives comes from intransitive entries. As we will elaborate in §7, we argue that both lexical reflexives and lexical reciprocals take one argument with a complex thematic role AgPt, where some properties of agents and patients are retained. This treatment encompasses the interpretation of lexical reflexives, where the active role of the agent is not necessarily retained, and of lexical reciprocals, where the active participation of both individuals is not always required. However, we do not delve into the question of how such lexical interpretations are truth-conditionally associated with the stem’s meaning. Rather, we use the semantic properties of English lexical reflexives to study the dissociation between lexical and grammatical processes in Romance.

In parallel to our observations on Romance reciprocals, lexical reflexives behave differently from grammatical reflexives in relation to the realization and the semantic effects of *se*. For example, with the transitive verb *descrever* ‘to describe’, the BP example in (65) gets the same interpretation associated with its grammatical reflexive English correlate.

(65)
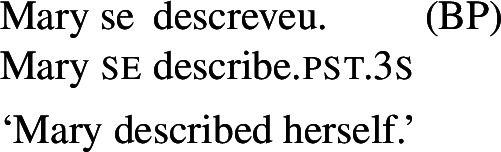

Now let us consider the two sentences in (66) below. The sentence with *se* in (66a) supports situations where Mary shaved her own self (a scenario that is in line with both plain and pseudo-reflexive readings), but it is also true in two kinds of exceptional situations. First, it is true in a situation where Mary volitionally went to the beautician for depilation; secondly, it is true in a situation where Mary shaved a statue of herself. These two kinds of situation are characteristic of lexical reflexivity and grammatical reflexivity respectively, as the translations of (66a) indicate. By contrast, example (66b) only supports a pseudo-reflexive reading: it is in line with situations where Mary shaved her own self or was volitionally shaved by a beautician, but it excludes the ‘proxy’ reading where Mary shaved a statue of herself.

(66)
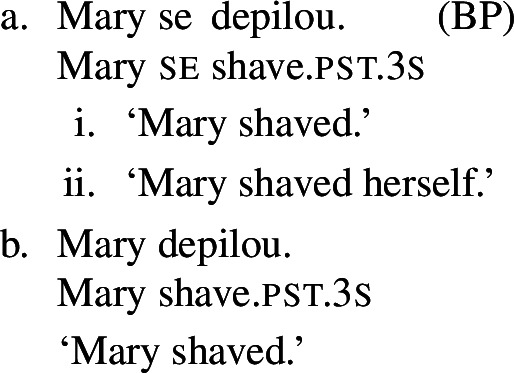

This pattern is fully parallel to what we observed with lexical and grammatical reciprocals (42).

Our second observation is also parallel to what we have already observed with lexical reciprocity. We saw that clauses with *se* and plural subjects are systematically ambiguous between reflexive and reciprocal interpretations, whereas clauses without *se* only get access to the inherent meaning of the verb’s entry. We now observe this also with lexical reflexive verbs. For example, let us consider sentence (67) below.

(67)
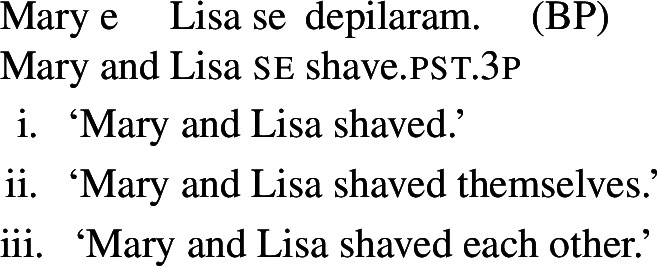

Just like its singular counterpart in (66a), sentence (67) above can be true in two kinds of exceptional situations. First, Mary and Lisa might have been shaved by someone else, but on their own accord; this is the kind of situation that characterizes the pseudo-reflexive reading. Second, each of Mary and Lisa might have shaved a statue of herself; this kind of situation falls under the plain reflexive reading. Additionally, like all sentences with *se* and a verb with a transitive entry, (67) also gets a plain reciprocal reading where Mary and Lisa shaved each other. Now let us consider sentence (68) below, where *se* is omitted:

(68)
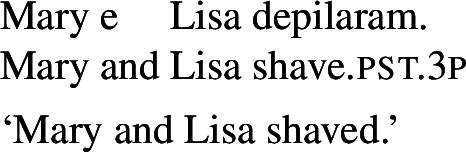

The sentence (68) is true as long as each individual referred to by the subject is a volitional patient of the action described by the verb (e.g., if each of Mary and Lisa shaved herself or went to the beautician). The form in (68) does not support a situation characteristic of grammatical reflexivity where Mary and Lisa each shaved a statue of herself, nor a reciprocal reading where they shaved each other. Accordingly, we characterize this sentence, like its singular counterpart (66b), as having an unambiguously pseudo-reflexive reading.

#### An intermediate summary

The data of Sects. 3 to 5 support the following generalizations on *se* and on its interactions with reflexive and reciprocal interpretations:

(69) **SE generalizations – lexical reciprocity and reflexivity**:
  - *Se*-clauses without an additional reciprocal or reflexive item allow both plain reflexivity and plain reciprocity.
  - Certain transitive verbs can appear in sentences without *se* or any other item expressing reflexivity or reciprocity. The interpretation of such sentences is unambiguously pseudo-reciprocal with some verbs, and unambiguously pseudo-reflexive with others. Accordingly, we refer to these verbs as *lexical reciprocals*/*reflexives*.
  - The pseudo-reciprocal/reflexive interpretation of these verbs is also retained when they appear with an overt *se*, on top of the plain reciprocal and reflexive interpretations that appear with all transitive verbs.

## Grammatical reciprocity and reflexivity without *se*

So far, we have proposed that reflexivity and reciprocity can be lexically expressed in Romance languages. This proposal is most easily supported by cases in which these interpretations appear while *se* is omitted. In this section, we will see that also grammatical arity-reducing operations can take place without *se*. This phenomenon uniformly occurs with all transitive verbs in environments that allow *se* omission, provided that they appear together with an overt reflexive/reciprocal element. As we will show, Romance reflexive/reciprocal pronominals and adverbials (like BP *si mesmo* ‘himself’ or Spanish *mutuamente* ‘mutually’) disambiguate the interpretation of *se*-clauses, ridding them of the reflexivity/reciprocity polysemy. Additionally, with lexical reflexives and reciprocals, such elements also remove the lexical pseudo-reflexive or pseudo-reciprocal reading.

The facts presented in this section, together with the generalization on lexical reciprocals and reflexives in (69), will lead to the theoretical picture proposed in Sect. 7: the reflexive and reciprocal items discussed below will be treated as semantic operators, and the clitic *se* as an arity-reduction marker that licenses reciprocal/reflexive interpretations, but does not carry an independent meaning of its own.

### Overt reciprocal elements

Overt reciprocal elements include adverbials like Italian *a vicenda* ‘mutually, in turns’ and Spanish *mutuamente* ‘mutually’, as well as pronominal elements like BP *um o outro* ‘one another’ and Catalan *l’un a l’altre* ‘one another’. These elements have three different functions that are relevant for our study.

First, when they appear with *se* they remove the reflexivity/reciprocity ambiguity, leading to a reciprocal interpretation. For example, the Italian clause in (70) can receive either a reflexive or a reciprocal reading, whereas only the latter is accessible in the presence of *a vicenda* in (71).

(70)

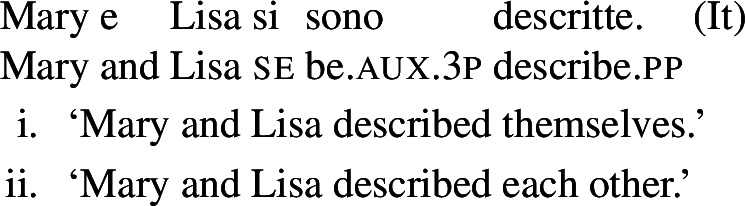

(71)

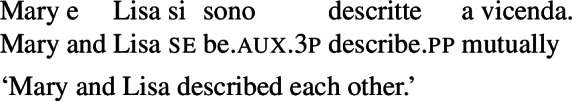

Second, overt reciprocal elements also remove the pseudo-reciprocal reading that appears with *se* and lexical reciprocals, leaving plain reciprocity as the only reading. For example, let us first consider the following example, without any overt reciprocal element:

(72)
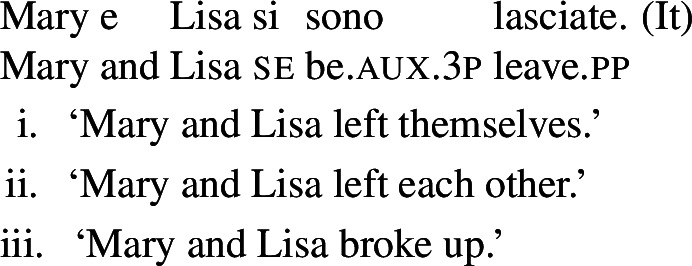

In sentence (72), on top of a less accessible plain reflexive reading (72i), the predicate *lasciare* ‘leave/break up’ leads to two prominent interpretations: one that is here paraphrased using a reciprocated transitive entry ‘leave’ (72ii), and another using the pseudo-reciprocal meaning ‘break up’ (72iii). The last reading does not entail two unidirectional relations, as the relationship could be unilaterally terminated by one individual. Thus, sentence (72) is considered true in a scenario where Mary terminated the relationship with Lisa, while Lisa was left heartbroken. When we add the element *a vicenda*, this pseudo-reciprocal reading disappears. Consider for instance what happens when *a vicenda* is added to sentence (72):

(73)
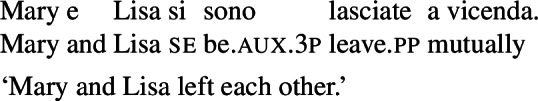

Unlike (72), sentence (73) cannot be accepted if the relationship between Mary and Lisa was unilaterally terminated: for the sentence to be true, each of the two people must have left the other. Thus, in (73) the adverbial *a vicenda* disambiguates (72) and only allows the plain reciprocal reading.

Third, in constructions where *se* can be omitted (Sect. 3), overt reciprocal expressios derive reciprocity without *se* for all transitive verbs. For example, in the Italian causative in (74) below, grammatical reciprocity is possible with *a vicenda*, but without *se*:

(74)
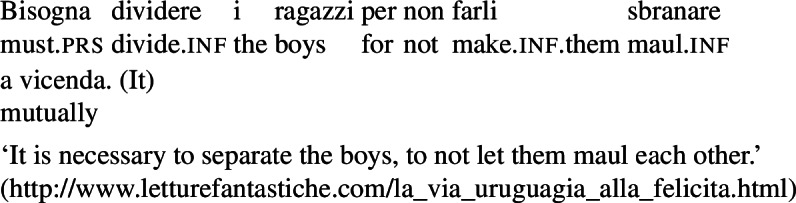

Similarly, BP transitive verbs can lead to reciprocity without *se* in finite clauses where the adverbial *um o outro* occurs.^22^ For example, let us consider sentence (75):

(75)
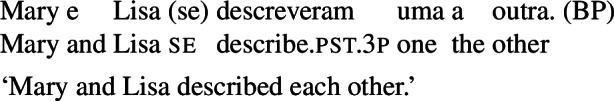

Sentence (75) unambiguously has the plain reciprocal interpretation where Mary described Lisa and Lisa described Mary. Due to the presence of the reciprocal item *um o outro*, and in contrast with sentence (20) above, the clitic *se* in (75) is only optional. The same observation extends to Spanish absolute constructions, where *mutuamente* ‘mutually’ can lead to a reciprocal interpretation without *se* with any transitive verb. For example:

(76)

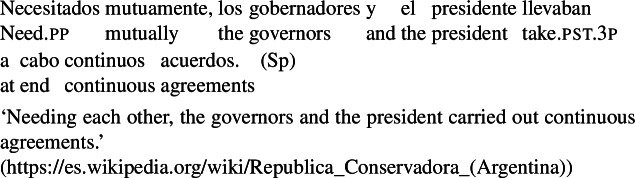

### Overt reflexive elements

Similar observations hold for reflexive elements like the pronominals BP *si mesmo* ‘himself’ in BP, *si mismo* ‘himself’ in Spanish, and *si mateix* in Catalan.^23^ First, these items disambiguate *se*-clauses by eliminating the reciprocal reading. For instance, while the BP sentence (77) shows the familiar reflexivity/reciprocity polysemy, (78) can only be interpreted reflexively.

(77)

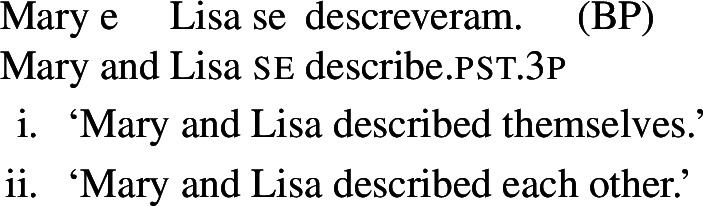

(78)

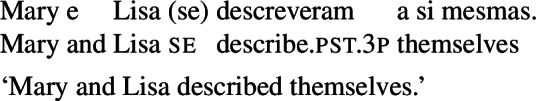

Second, when they appear with lexical reflexive predicates, overt reflexive elements disallow the pseudo-reflexive interpretation. For instance, the BP sentence (79a) (=(66a)) supports a pseudo-reflexive interpretation where Mary was volitionally shaved by a beautician, whereas (79b) requires that Mary shaved herself.

(79)

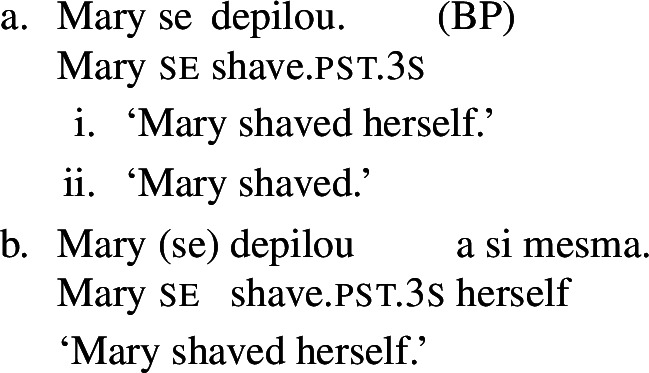

Finally, overt reflexives are allowed without *se* in the same environments that allow lexical reciprocity/reflexivity without *se*. Such constructions receive plain reflexive interpretations. For example, in the *si mesmo* finite clause in (80) below, *se* can either appear or not, and the interpretation is plain reflexive either way:

(80)

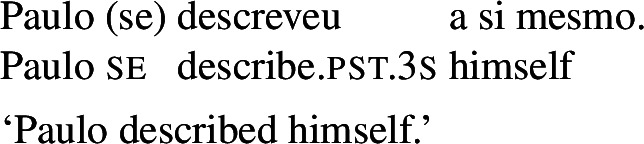

We summarize this section using the following generalizations about *se* and overt reciprocal and reflexive items, which complement the generalizations in (69):

(81) **SE generalizations – overt reciprocity and reflexivity**:
  - Clauses (with or without *se*) containing a reciprocal (reflexive) item are unambiguously reciprocal (reflexive, respectively).
  - When a lexical reciprocal or reflexive predicate appears with a reciprocal (reflexive) item, it only shows plain reciprocity (reflexivity, respectively), but no pseudo-reciprocity or pseudo-reflexivity.
  - The same environments that support pseudo-reciprocity and pseudo-reflexivity without *se* also support plain reciprocity (reflexivity) without *se*, provided that they contain an overt reciprocal (reflexive, respectively) item.

## *Se* as a functional head projection

This section proposes a unified analysis of generalizations (69) and (81), focusing on the syntactic-semantic role of *se* with lexical and grammatical reciprocity/reflexivity. We follow Labelle (2008) in assuming that *se* is a Voice head projection. However, we diverge from Labelle’s proposal that *se* has a direct contribution to reflexive or reciprocal meanings as an operator that binds external and internal arguments. Instead, we propose that *se* is a marker *à la* Reinhart and Reuland (1993), which marks the VP as reflexive/reciprocal, without providing the reflexive/reciprocal meaning itself. In our analysis, arity-reducing operators may be overt (like the reflexive and reciprocal items discussed in Sect. 6) or they can operate covertly, licensed by *se*.

Labelle (2008) proposes that French *se* is responsible for reflexive/reciprocal interpretations when it combines with transitive predicates, but it is semantically redundant with predicates that already have a reflexive/reciprocal reading. To illustrate Labelle’s account, let us consider the following French examples:

(82)

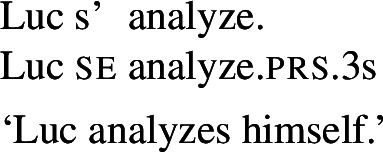

(83)
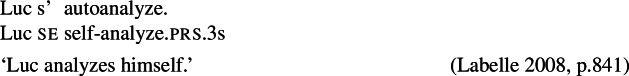

Labelle’s main point about these examples is reminiscent of the facts that we discussed in Sect. 6: French *se* leads to reflexivity (or reciprocity) with ordinary transitive verbs (82), but it also appears with verbs whose reflexive (or reciprocal) meaning comes from an additional element, e.g., *auto-* in (83). Labelle’s syntactic-semantic analysis of examples like (82) and (83) is given in (84) and (85) below.^24^

(84) Following Labelle (2008, (31), p.844):

(85) Labelle (2008, (32), p.844):

Labelle’s analysis relies on Kratzer’s (1996) neo-Davidsonian semantics. In Labelle’s use of Kratzer’s proposal, *se* is a functional head that introduces the external argument (*x* in the component Agent(e,x)) and binds it to the internal argument of the verb (*x* in P(e,x)). With transitive verbs as in (84), *se* introduces the event’s agent as an external argument, binding it to the internal argument. By contrast, verbs that are prefixed by *auto-* or *entre-*, like *autoanalyzer* in (85), already contain an external argument variable in their denotation. In such cases, *se* has the same meaning as with transitive verbs. However, Labelle’s neo-Davidsonian assumption is that the Agent operator is a (possibly partial) function on events, which maps every event to a *unique* agent (if any). This assumption makes sure that when the Agent operator is introduced twice for the same event (e.g., by the *auto-* prefix and by *se*) it binds the same entity to the external argument. Although *se* is semantically redundant in such cases, Labelle (2008) notes that it is nonetheless obligatory for the grammaticality of French sentences like (83). Labelle explains this requirement by assuming that *se* is needed to ensure a coherent interpretation: it prevents Active Voice from introducing a distinct variable for the subject. The idea in Labelle (2008) is that in the absence of *se*, Active Voice would occupy the Voice head and it would introduce an external argument which would not be bound to the internal argument of the VP. Labelle’s assumption (which we question below) is that this would lead to an incoherent interpretation where there are two distinct unsaturated external positions for one and the same subject, a situation that would violate the Theta-criterion.

Although Labelle’s analysis is the starting point for our treatment of *se*, it presents some incompatibilities with the observations in previous sections, and it raises some questions about its own merits. One problem is a problem of generality. Labelle’s analysis relies on the assumption that whenever *se* appears, the verb has an internal argument. Labelle’s semantic analysis works for verbs like *autoanalyzer*, which are the outcome of a productive prefixation process. However, the analysis should be extended to capture the semantics of the lexical reciprocals we covered in §4.3 (as well as lexical reflexives §5). As we saw, the inherent reading of many lexical reciprocals and reflexives is not in line with multiple thematic roles. Thus, the pseudo-reciprocal/reflexive interpretations of lexical reciprocals like ‘hug’ or lexical reflexives like ‘wash’ cannot be derived by a standard binding mechanism like the one Labelle proposes for *auto-* and *entre-* prefixation. Another problem of generality appears with Labelle’s assumption that *se* is uniformly needed with transitive verbs to co-index the internal argument and the external argument of VPs. As we saw, there are many examples of transitive verbs in Romance where *se* does not appear, and a reciprocal or reflexive interpretation emerges due to the presence of another reciprocal/reflexive item. Such cases are not addressed in Labelle’s account, where the presence of *se* is considered as a ubiquitous requirement to ensure reflexive/reciprocal interpretations. Another problem lies in the semantic motivations for the presence of *se*. As mentioned earlier, Labelle proposed that *se* is necessary to prevent Active Voice from occupying the Voice head, which she assumes would result in the introduction of two different external arguments for verbs like *autoanalyzer* (one introduces by *auto*-, one by Active Voice). As we mentioned above, the identification of the two agent arguments in Labelle’s analysis (85) is a basic assumption of the neo-Davidsonian approach, where *Agent* is treated as a function from events to entities. Due to this property of the semantic framework that Labelle relies on, it is unclear what meaning she proposes for Active Voice that would introduce a different agent to the event from the one introduced using *auto-*.^25^

To overcome these issues, we propose an alternative explanation for the required presence of *se*, where it is not directly responsible for reflexive and reciprocal interpretations. We follow Labelle’s proposal that *se* is a Voice head projection, but we argue that *se* never contributes to reflexive or reciprocal meanings all by itself. Instead, we propose that *se* combines with VPs that already have a reflexive/reciprocal interpretation: either a lexical interpretation due to the intransitive meaning of the verb stem, or a grammatical interpretation derived by a reflexive/reciprocal operator. We propose that such operators can be introduced overtly (e.g., as pronouns or adverbials) or covertly, as operators that are responsible for the semantics of arity-reduction. Overt operators perform the necessary ‘marking’ of a VP headed by a transitive verb as being reflexive/reciprocal, whereas covert operators do not. We propose that it is only in such cases, where such R(eflexive/reciprocal)-marking is missing, that the introduction of *se* is necessary to make the reflexive/reciprocal semantics correspond with morpho-syntax. We propose that the variability in the presence of *se* across different Romance languages emerges due to different syntactic restrictions on the presence of this element.

In more detail, we analyze the appearance of *se* as relying on four different factors:

(i) **Types**: We follow the typed meaning that is assumed by Kratzer and Labelle for Active Voice as restated in (86) below: an operator that takes predicates over events (type *st*) and adds to them an external argument, which leads to a predicate of type *e*(*st*).^26^ We propose that Active Voice is ruled out with reflexive/reciprocal VPs due to a simple type mismatch. We assume that such VPs already contain an external argument variable, which is either part of the lexical entry or introduced by a grammatical operator. Thus, reflexive and reciprocal verb entries are assigned the lexical type *e(st)*. This is in contrast to other intransitive verbs that are assumed to be of the neo-Davidsonian type *st*. This contrast is illustrated in (87) below. The intransitive reflexive/reciprocal entry of verbs like *shave/hug* is analyzed as a predicate ‘shave_1_’/‘hug_1_’ that has an external argument with the thematic role ‘AgPt’. This thematic role shows both agent-like and patient-like semantic properties of the corresponding transitive entry, which is denoted ‘shave_2_’/‘hug_2_’. Further, overt reciprocal and reflexive items like *si mesmo* (BP, ‘himself’, (88)) or *um o outro* (BP, ‘each other’) turn transitive predicates into predicates with an external argument that bears the thematic roles of both Agent and Patient. For the clitic *se* we propose the typed function in (89): the (semantically void) identity function on two-place predicates over entities and events. As a result of these types and meanings, lexical reflexives/reciprocals and transitive verbs with a reflexive/reciprocal operator can combine with *se*, but not with Active Voice. Conversely, non-reflexive/non-reciprocal intransitive verbs like ‘laugh’ can combine with Active Voice but not with *se*. A major difference between the two kinds of VPs is that with simple intransitives like ‘laugh’, an additional operation is required in order to add their external argument, whereas reflexive/reciprocal VPs contain the external argument as part of the lexical meaning of the VP.
  (86)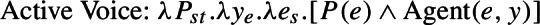  (87)
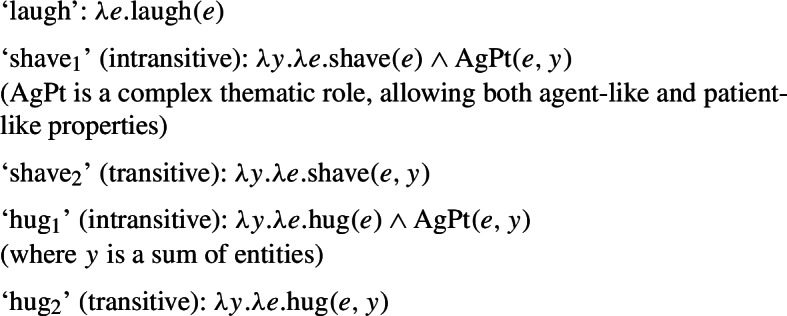
  (88)
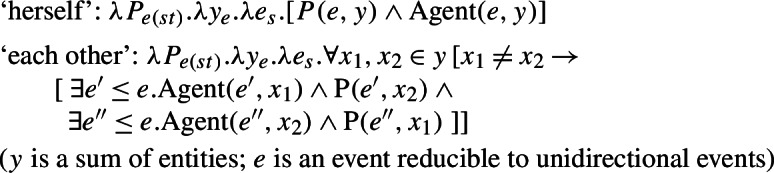
  (89)
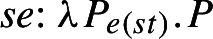
(ii) **Covert operators**: As we mentioned above, we assume that *se* licenses covert reciprocal and reflexive operators, which have the same meanings as ‘herself’ and ‘each other’ in (88).^27^
  (90)
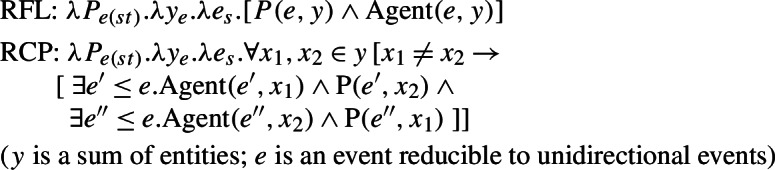
(iii) **R-marking**: Like all overt reflexive and reciprocal items in Romance, *se* has the function of syntactically ‘marking’ reflexive/reciprocal predicates, in the sense of Reinhart and Reuland’s (1993) analysis of Conditions A and B. We implement Reinhart and Reuland’s proposal as follows:
  (91) **Condition A**: An R-marked predicate has a reflexive/reciprocal interpretation.^28^
    **Condition B**: Any reflexive/reciprocal interpretation of a predicate requires R-marking.
  Specifically, Condition A requires that any transitive verb appearing with *se* or an overt reflexive/reciprocal item has a reflexive/reciprocal interpretation. We have seen that this requirement systematically holds in Romance languages. Condition B requires that any verb that is interpreted reflexively/reciprocally must be R-marked. Like Reinhart and Reuland, we assume that this R-marking can come from *se*, from an overt reflexive/reciprocal item, or from a reflexive/reciprocal intransitive in the lexicon. Thus, reflexive/reciprocal intransitives satisfy Condition B even when they are not accompanied by any overt R-marker like *se* or an overt reflexive/reciprocal item. In the absence of a lexical reflexive/reciprocal entry, reflexive and reciprocal interpretations cannot emerge from the verb alone. Thus, for Condition B to be satisfied in that situation, transitive predicates must be marked by an overt reflexive pronominal and/or by *se*.
(iv) **Syntactic construction**: We observe that there are syntactic constructions where *se* may or must be omitted. We take this as a distributional fact about *se*, and we consider here three types of syntactic environments in Romance in relation to the presence/absence of *se* with reflexive/reciprocal interpretations:
  **+SE**, where *se* is obligatory (It/Sp/Cat finite clauses)
  **−SE**, where *se* is disallowed (It causatives, Sp/Cat absolutes)
  **±SE**, where *se* is optional (BP finite clauses, Sp/Cat causatives).
  We hypothesize that the possible omission of *se* correlates to the presence/absence of a Voice projection to host this element. For causatives, this is in line with the existing proposal that Romance causative complements do not project functional layers (Ciutescu 2013; Pitteroff and Campanini 2013), and generally lack an external argument (Labelle 2017). For BP finite clauses, a connection between *se*-omission and absence of a Voice head is proposed in Carvalho (2018). This hypothesis raises questions with respect to syntactic parameters underlying constructions with or without *se*, and with respect to the cross-linguistic variation within Romance. We defer these questions to future work. For our present purposes, it is sufficient to observe that the requirements for the presence of *se* vary across languages and constructions. The hypothesis that availability of Voice is the key for this variation is convenient for our present goals, but it requires further study and is not part of our core proposal.

Let us now exemplify the working of this fourfold proposal. We start with intransitive predicates that do not receive a reflexive or reciprocal interpretation, like ‘laugh’ (92). For such verbs, the external argument is introduced in Voice using the denotation of Active Voice, as demonstrated in (93).

(92)
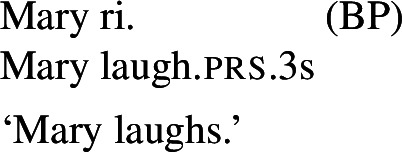

(93)
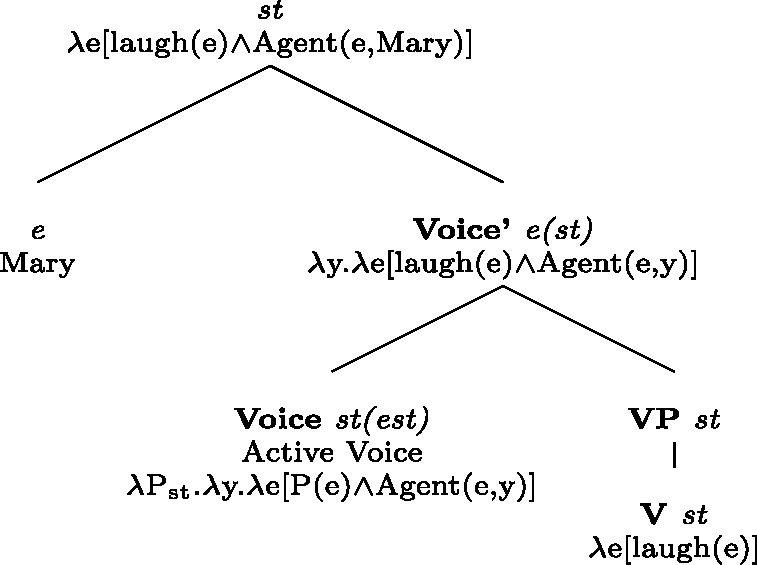

By contrast, VPs that are interpreted as reflexive/reciprocal are treated as containing an Agent variable. Let us first consider lexical reflexives/reciprocals as in (94). With VPs headed by such verbs, the external argument variable is already part of the lexical entry, which contains the complex thematic role AgPt (95). The predicate is of type *e(st)*, and it cannot combine with Active Voice. In +SE syntactic environments, the predicate appears with *se*. This element does not introduce the external argument and does not have any reflexive or reciprocal semantic content: it merely marks that the VP is reflexive/reciprocal, by stating that no Agent variable is introduced in Voice. Note that because the verb has a lexical reflexive entry, which is assumed to be an R-marker, Condition B is also satisfied without *se*. The presence of *se* merely depends on the syntactic requirements of the clause: it is obligatory in +SE constructions, but not in –SE or ±SE environments, like BP finite clauses (94). In either case, the interpretation of lexical reflexives/reciprocals originates from the verb stem.

(94)

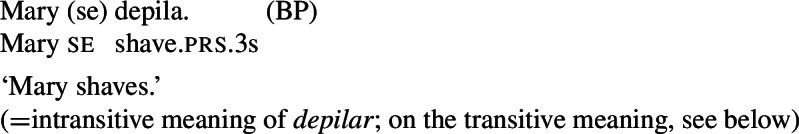

(95)

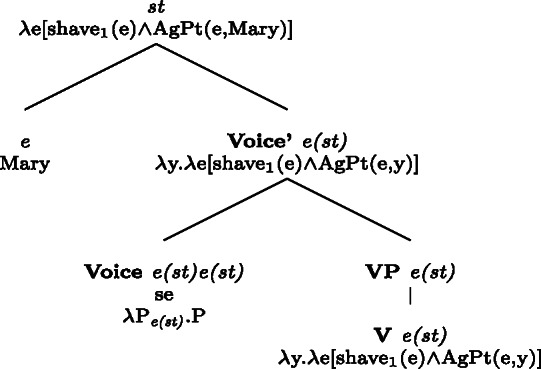

The variant of (94) with *se* also supports the grammatical strategy, which applies with the transitive meaning of *depilar*. We propose that this polysemy of *depilar* leads to structural ambiguity with the *se* variant of (94), which we examine more closely in (96) below.^29^ The reading in (96i.) is the same that we analyze in (95) above as stemming from the lexical reflexive entry ‘depilar_1_’. The reading in (96ii.) is due to the transitive entry ‘depilar_2_’, here reflexivized using the covert reflexive operator RFL (97). This interpretation requires the presence of *se* to satisfy Condition B, because R-marking is performed neither by the transitive entry, nor by the covert RFL operator. In this case, *se* is required for R-marking (although by itself it does not provide the reflexive interpretation), whereas the covert RFL operator is responsible for the reflexive interpretation (but it does not provide R-marking). Thus, although sentence (96) is treated as ambiguous between lexical reflexivity and grammatical reflexivity, our analysis unifies the role of *se* with the two strategies.

(96)

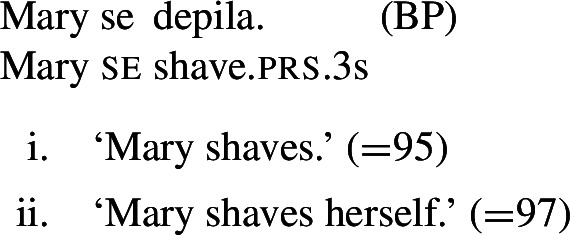

(97)

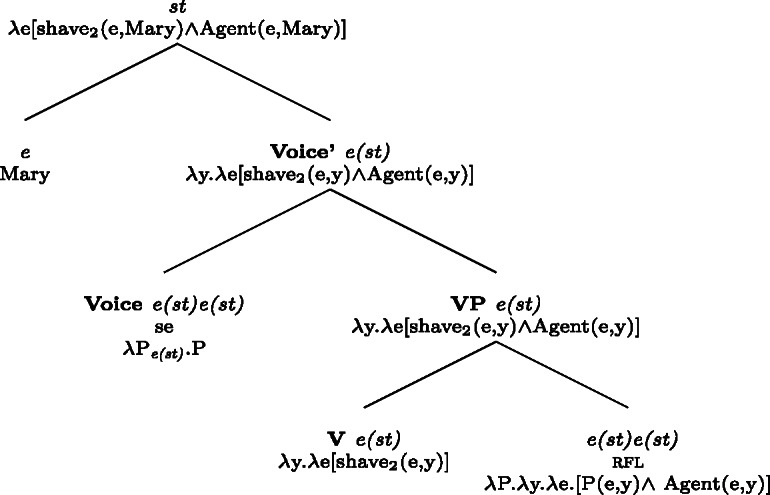

Let us now analyze grammatical strategies more closely. Recall that grammatical reflexivity and reciprocity can be realized in three different configurations; they are exemplified in (98) below for BP, using the unambiguously transitive predicate ‘describe’. We propose that plain reflexive/reciprocal interpretations consistently come from reflexive/reciprocal operators that can be realized overtly (e.g., BP *si mesmo*/*um o outro*) or covertly (RFL/RCP operators). Overt and covert operators have the same meaning; the only difference is that overt operators can satisfy Condition B, whereas covert operators cannot because they are not morpho-phonologically realized. Thus, overt operators can operate without *se*, whereas covert operators cannot lead to reflexive/reciprocal readings all by themselves. Both overt and covert reflexive/reciprocal operators introduce the external argument variable and assign it the thematic roles of both Agent and Patient (99). Just like in the case of the lexical strategy, this results in a VP of type *e(st)* that cannot combine with Active Voice, but can combine with *se* whenever this element is allowed or required. Once again, *se* does not have any reflexive semantic content (99).

(98)

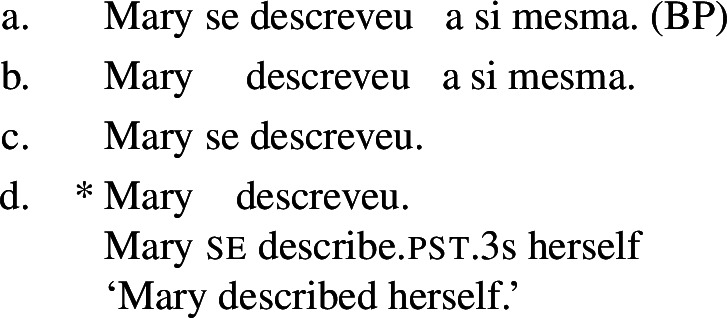

(99)

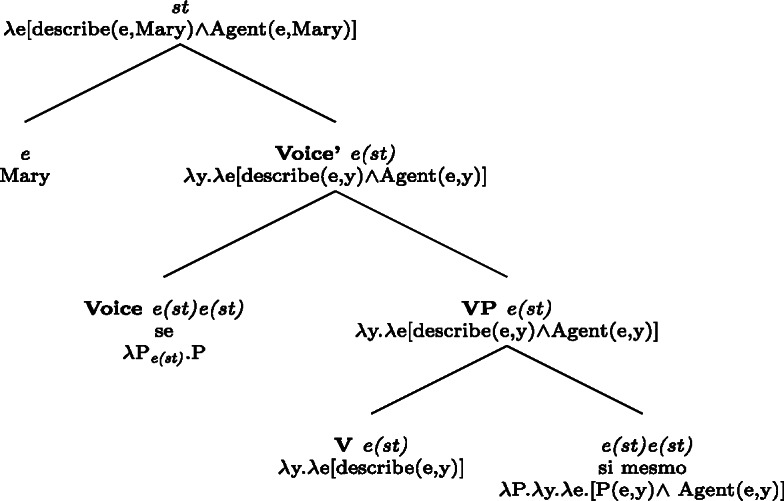

In sentence (98a), all the components of the representation in (99) are overtly realized. The clause in (98b) is compositionally analyzed like (98a), but without *se*. This is possible because the overt operator already satisfies Condition B, so *se* can be omitted in –SE and ±SE constructions, like BP finite clauses (98b). By contrast, in (98c) the operator responsible for the reflexive interpretation is covert. For this reason, *se* is required to satisfy Condition B: the absence of *se* results in ungrammaticality (98d), or in the unavailability of reflexive/reciprocal readings (e.g., in Italian causatives (26), §3.2).

Let us conclude by discussing the familiar ambiguity between reflexivity and reciprocity in *se*-clauses. The clitic *se* licenses both the covert reflexive operator RFL and the covert reciprocal operator RCP. For this reason, *se*-clauses with a plural subject support both reflexive and reciprocal interpretations, and can only be disambiguated by further contextual information. Furthermore, if the verb has a lexical reflexive/reciprocal entry, the clause receives an additional interpretation coming from the intransitive verb stem. Throughout the paper, we have explored this three-way ambiguity in sentences like (42a) and (67). (67) is repeated below as (100). With our proposed analysis, we can now examine the emergence of these three readings. The pseudo-reflexive reading in (100i.) is due to the inherent meaning of the intransitive ‘depilar_1_’, derived as in (94). The plain reflexive reading in (100ii.) and the plain reciprocal reading in (100iii.) contain the transitive verb ‘depilar_2_’, as in (97). The interpretation in (100ii.) is due to the covert reflexive operator RFL, whereas (100iii.) is due to the covert reciprocal operator RCP. As previously seen, only the pseudo-reflexive interpretation in (100i.) survives if *se* is omitted: the interpretations in (100ii.) and (100iii.) require *se* to license the RFL and RCP operators.

(100)

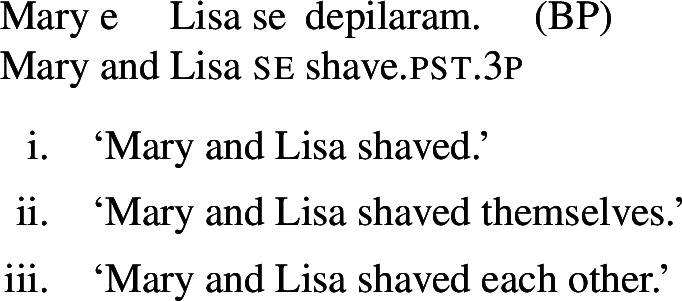

## Conclusions

Reciprocal and reflexive interpretations result from lexical and grammatical strategies that have been argued to exist in all languages. In this paper, we studied the case of Romance languages, where the two strategies are not always morpho-syntactically distinct. In these languages, many environments invariably require the element *se* for expressing a reciprocal meaning or a reflexive meaning. While this is a considerable obstacle for characterizing reciprocity and reflexivity in Romance semantics, in this paper we have aimed to show that the challenge is not unsurmountable. Romance lexical reciprocals and reflexives can be fruitfully studied based on properties that cross-linguistically characterize this class of predicates. We focused on Italian, Brazilian Portuguese, Spanish, and Catalan. In these four language, we identified a class of verbs that, in constructions that vary between languages, express reciprocity without *se* or other reciprocal elements, without giving rise to a reflexivity/reciprocity ambiguity. We showed that systematic semantic characteristics of such cases give substantial support to the claim that Romance lexicons contain reciprocal and reflexive verbs, whose meanings are fairly stable across languages.

Moving on to the role of *se* in the semantic derivation, we pointed out that in the presence of an overt reciprocity/reflexivity operator, *se* can be omitted in precisely the same environments where it is not required with lexical reciprocals/reflexives. These data go against accounts of *se* as operating directly on the verbal valency, and support the treatment of *se* as a functional head projection, along the lines of Labelle (2008). We extended Labelle’s analysis, arguing that *se* never has any reciprocal or reflexive semantics, although it has a central role in licensing reflexivity and reciprocity in the spirit of Reinhart and Reuland (1993).

The distribution of *se* clitics varies remarkably across different Romance languages. Yet, we believe that the current work offers a unifying perspective on some of the key challenges by showing the ways in which syntactic projections, semantic types, binding conditions, and covert operators interact in relation to lexical and grammatical functions. Further, we believe that the data we presented and the theoretical perspective we proposed may also prove useful for studying non-Romance languages whose reciprocal markers are comparable to Romance, e.g., German (Everaert 1986; Gast and Haas 2008), Icelandic (Wood 2014), Serbo-Croatian (Marelj 2004), Polish, and Slovenian (Rivero and Sheppard 2003; Wiemer 2007). Future studies may also reveal a contrast between the grammatical and lexical strategies beyond the Indo-European family, in other languages without clear distinctions between these two strategies, such as Lingala and Kanuri (Kemmer 1993). Such a larger language sample may allow us to substantially test the hypothesis (cf. Haspelmath (2007)) that all natural lexicons contain reciprocal and reflexive verbs. Furthermore, the set of concepts that these verbs refer to may possibly be governed by universal principles. Whether these ideas can be substantiated remains to be seen, but we hope that the present paper has highlighted possible directions for future studies.

## Appendix A

**Table 3:** Brazilian Portuguese

verb	finite clause	causative	discontinuous	group NP
*abraçar* ‘to hug’	*X e Y (se) abraçaram* ‘X and Y hugged’	*Eu fiz X e Y (se) abraçarem* ‘I caused X and Y to hug’	*X (se) abraçou com Y* ‘X and Y hugged’	*O time (se) abraçou* ‘The team hugged’
*beijar* ‘to kiss’	*X e Y (se) beijaram* ‘X and Y kissed’	*Eu fiz X e Y (se) beijarem* ‘I caused X and Y to kiss’	*X (se) beijou com Y* ‘X kissed with Y’	*O casal (se) beijou* ‘The couple kissed’
*casar* ‘to marry’	*X e Y (se) casaram* ‘X and Y got married’	*Eu fiz X e Y (se) casarem* ‘I caused X and Y to get married’	*X (se) casou com Y* ‘X got married with Y’	*O par (se) casou* ‘The pair got married’
*consultar* ‘to consult/confer’	*X e Y *(se) consultam* ‘X and Y confer/consult each other’	*Eu fiz X e Y *(se) consultarem* ‘I caused X and Y to confer’	*X (se) consulta com Y* ‘X confers with Y’	*O time *(se) consulta* ‘The team confers’
*cumprimentar* ‘to greet’	*X e Y (se) cumprimentaram* ‘X and Y greeted each other’	*Eu fiz X e Y (se) cumprimentarem* ‘I caused X and Y to greet each other’	*?X (se) cumprimentou com Y* ‘X and Y greeted each other’	*O time *(se) cumprimentou* ‘The team greeted each other’
*encontrar* ‘to find/meet’	*X e Y *(se) encontram* ‘X and Y meet/find each other’	*Eu fiz X e Y (se) encontrarem* ‘I caused X and Y to meet’	*X (se) encontrou com Y* ‘X met with Y’	*O time *(se) encontrou* ‘The team met’
*namorar* ‘to date/be in a relationship with’	*X e Y (se) namoram* ‘X and Y are partners’	*Eu fiz X e Y (se) namorarem* ‘I caused X and Y to be partners’	*X *(se) namora com Y* ‘X and Y are partners’	*O casal (se) namora* ‘The couple is in a relationship’

## Appendix B

**Table 4:** Catalan

verb	finite clause	causative	absolute	discontinuous	group NP
*abraçar* ‘to hug’	*X i Y *(s’) abraçen* ‘X and Y hug’	*He fet abraçar a X i Y* ‘I caused X and Y to hug’	*Abraçats X i Y surten* ‘After hugging, X and Y left’	*X s’abraça amb Y* ‘X and Y hug’	*L’equip s’abraça* ‘The team hugs’
*casar* ‘to marry’	*X e Y *(es) casen* ‘X and Y get married’	*He fet casar a X y Y* ‘I caused X and Y to get married’	*Casats X i Y surten* ‘After getting married, X and Y left’	*X es casa amb Y* ‘X gets married with Y’	*La parella es casa* ‘The couple gets married’
*consultar* ‘to consult/confer’	*X i Y *(es) consultan* ‘X and Y confer/consult each other’	*?He fet consultar a X y Y* ‘I caused X and Y to be consulted’	*?Consultats X i Y surten* ‘After conferring, X and Y left’	*?X se consulta amb Y* ‘X confers with Y’	*L’equip es consulta* ‘The team confers’
*deixar* ‘to leave/break up’	*X i Y *(es) deixen* ‘X and Y break up/leave each other’	*He fet deixar a X i Y* ‘I caused X and Y to break up’	*Deixats X i Y surten* ‘After breaking up, X and Y left’	*X es deixa amb Y* ‘X breaks up with Y’	*La parella es deixa* ‘The couple breaks up’
*petonejar* ‘to kiss’	*X i Y *(es) petonejen* ‘X and Y kiss’	*He fet petonejar a X i Y* ‘I caused X and Y to kiss’	*Petonejats X i Y surten* ‘After kissing, X and Y left’	*X es petoneja amb Y* ‘X kisses with Y’	*L’equip es petoneja* ‘The team kisses’
*topar* ‘to bump into/run into’	*X i Y *(es) topen* ‘X and Y bump into each other’	*?He fet topar a X i Y* ‘I caused X and Y to bump into each other’	*?Topats X i Y surten* ‘After bumping into each other, X and Y left’	*X es topa amb Y* ‘X and Y bump into each other’	*L’equip es topa* ‘The members of the team bump into each other’
*trobar* ‘to find/meet’	*X y Y *(es) troben* ‘X and Y find each other/meet’	*He fet trobar a X i Y* ‘I caused X and Y to meet’	*?Trobats X y Y salieron* ‘After being found, X and Y left’	*X es troba amb Y* ‘X meets with Y’	*L’equip es troba* ‘The team meets’

## Appendix C

**Table 5:** Italian

verb	*se* in finite clauses	causative	discontinuous	group NPs
*abbracciare* ‘to hug’	*X e Y *(si) abbracciano* ‘X and Y hug’	*Ho fatto abbracciare X e Y* ‘I caused X and Y to hug’	*X si abbraccia con Y* ‘X and Y hug’	*La squadra si abbraccia* ‘The team hugs’
*baciare* ‘to kiss’	*X e Y *(si) baciano* ‘X and Y kiss’	*Ho fatto baciare X e Y* ‘I caused X and Y to kiss’	*X si bacia con Y* ‘X kissed with Y’	*La coppia si sta baciando* ‘The couple is kissing’
*conoscere* ‘to know (of)’	*X e Y *(si) conoscono* ‘X and Y know (of) each other’	*Ho fatto conoscere X e Y* ‘I caused X and Y to get to know each other’	*?X si conosce con Y* ‘X and Y are acquainted/know each other’	*La famiglia si conosce bene* ‘The members of the family know each other well’
*consultare* ‘to consult/confer’	*X e Y *(si) consultano* ‘X and Y confer/consult each other’	*Ho fatto consultare X e Y* ‘I caused X and Y to confer’	*X si consulta con Y* ‘X confers with Y’	*La squadra si consulta* ‘The team confers’
*frequentare* ‘to date’	*X e Y *(si) frequentano* ‘X and Y date’	*Ho fatto frequentare X e Y* ‘I caused X and Y to date’	*X si frequenta con Y* ‘X is dating with Y’	*La coppia si frequenta* ‘The couple dates’
*incontrare* ‘to meet’	*X e Y *(si) incontrano* ‘X and Y meet’	*Ho fatto incontrare X e Y* ‘I caused X and Y to meet’	*X si incontra con Y* ‘X meets with Y’	*La squadra si incontra* ‘The team meets’
*incrociare* ‘to cross/run into’	*X e Y *(si) sono incrociati* ‘X and Y crossed each other/ran into each other’	*Ho fatto incrociare X e Y* ‘I caused X and Y to run into each other’	*X si è incrociato con Y* ‘X and Y ran into each other’	*La famiglia si è incrociata per caso* ‘The members of the family ran into each other accidentally’
*lasciare* ‘to leave/break up’	*X e Y *(si) lasciano* ‘X and Y break up/leave each other’	*Ho fatto lasciare X e Y* ‘I caused X and Y to break up’	*X si è lasciato con Y* ‘X broke up with Y’	*La coppia si è lasciata* ‘The couple broke up’
*sposare* ‘to marry’	*X e Y *(si) sposano* ‘X and Y get married’	*Ho fatto sposare X e Y* ‘I caused X and Y to get married’	*X si sposa con Y* ‘X gets married with Y’	*La coppia si sposa* ‘The couple gets married’
salutare ‘to greet’	*X e Y *(si) salutano* ‘X and Y greet each other’	*Ho fatto salutare X e Y* ‘I caused X and Y to greet each other’	*?X si saluta con Y* ‘X and Y greet each other’	*La famiglia si saluta* ‘The members of the family greet each other’
*trovare* ‘to find/meet’	*X e Y *(si) trovano* ‘X and Y find each other/meet’	*Ho fatto trovare X e Y* ‘I caused X and Y to meet’	*X si è trovato con Y* ‘X met with Y’	*La coppia si trova ogni settimana* ‘The couple meets every week’
*vedere* ‘to see/meet’	*X e Y *(si) vedono* ‘X and Y see each other/meet’	*?Ho fatto vedere X e Y* ‘I caused X and Y to meet’	*X si vede con Y* ‘X meets with Y’	*La coppia si vede ogni settimana* ‘The couple meets every week’

## Appendix D

**Table 6:** Spanish

verb	*se* in finite clauses	causative	absolute	discontinuous	group NPs
*abrazar* ‘to hug’	*X y X *(se) abrazan* ‘X and Y hug’	*Hice abrazar a X y Y* ‘I caused X and Y to hug’	*Abrazados X y Y salieron* ‘After hugging, X and Y left’	*X se abraza con Y* ‘X and Y hug’	*El equipo se abraza* ‘The team hugs’
*besar* ‘to kiss’	*X y Y *(se) besan* ‘X and Y kiss’	*Hice besar a X y Y* ‘I caused X and Y to kiss’	*?Besados X y Y salieron* ‘After kissing, X and Y left’	*X se besa con Y* ‘X kissed with Y’	*La pareja se besa* ‘The couple kisses’
*casar* ‘to marry’	*X y Y *(se) casan* ‘X and Y get married’	*Hice casar a X y Y* ‘I caused X and Y to get married’	*Casados X y Y salieron*‘After getting married, X and Y left’	*X se casa con Y* ‘X gets married with Y’	*La pareja se casa* ‘The couple gets married’
*consultar* ‘to consult/confer’	*X y Y *(se) consultan* ‘X and Y confer/consult each other’	*Hice consultar a X y Y* ‘I caused X and Y to confer’	*?Consultados X y Y salieron* ‘After conferring, X and Y left’	*X se consulta con Y* ‘X confers with Y’	*El equipo se consulta* ‘The team confers’
*cruzar* ‘to cross/run into’	*X y Y *(se) cruzaron* ‘X and Y crossed each other/ran into each other’	*Hice cruzar a X y Y* ‘I caused X and Y to run into each other’	*?Cruzados X y Y salieron* ‘After running into each other, X and Y left’	*X se cruzó con Y* ‘X and Y ran into each other’	*La familia se cruzó por casualidad* ‘The members of the family ran into each other accidentally’
*dejar* ‘to leave/break up’	*X y Y *(se) dejaron* ‘X and Y broke up/left each other’	*Hice dejar a X y Y* ‘I caused X and Y to break up’	*Dejados X y Y salieron* ‘After breaking up, X and Y left’	*X se dejó con Y* ‘X broke up with Y’	*La pareja se dejó* ‘The couple broke up’
*encontrar* ‘to find/meet’	*X y Y *(se) encuentran* ‘X and Y meet/find each other’	*Hice encontrar a X y Y* ‘I caused X and Y to meet’	*Encontrados X y Y salieron* ‘After meeting, X and Y left’	*X se encuentra con Y* ‘X meets with Y’	*El equipo se encuentra* ‘The team meets’
*ver* ‘to see/meet’	*X y Y *(se) ven* ‘X and Y see each other/meet’	*?Hice ver a X e Y* ‘I caused X and Y to be seen’	*?Vistos X y Y salieron* ‘After being seen, X and Y left’	*X se ve con Y* ‘X meets with Y’	*La pareja se ve cada semana* ‘The couple meets every week’

## Footnotes

1 Unless otherwise specified, judgments on most examples (in Italian, Spanish, Catalan, and Brazilian Portuguese) are based on the authors’ native intuitions. These judgments, as well as all judgments on examples in other languages, are supported by intuitions of native speaker consultants or from the literature.

2 Abbreviations used in this paper: prs = present tense, pst = past tense, pp = past participle, inf = infinitival, aux = auxiliary, *n*s = *n* person singular, *n*p = *n* person plural, nact = non-active voice, dom = differential object marking, refl = reflexive, rec = reciprocal, act = active.

3 These properties will be closely examined in relation to Romance lexical reciprocals in §4.3 and §4.4.

4 These assumptions will be further discussed in §7, along with a more comprehensive overview of Labelle (2008).

5 These predicates include, but are not limited to, verbs bearing the Latin reciprocal/sociative prefix *com-* (Zaliznjak and Shmelev 2007), as in *competere* ‘to compete’ or *convivere* ‘to cohabit’ in Italian.

6 Different BP speakers have different judgements on the acceptability of sentences without *se* like (21b). However, all BP speakers that were consulted accepted reciprocity without *se* for some or other verbs of the list in (29) below. It is well-known that BP has a large number of different dialects according to geographic and sociolinguistic parameters (Cardoso et al. 2011). This variation has been also shown to affect the distribution of *se* (Teixeira and da Silva 2019). However, this variability does not threaten our general proposal. We claim that if a BP speaker ever accepts a reciprocal interpretation without *se* (and without another overt reciprocal element), then she accepts it with at least some of the verbs that cross-linguistically have intransitive reciprocal meanings (e.g., ‘kiss’ or ‘hug’). Conversely, verbs like ‘describe’, which cross-linguistically have only a transitive entry, uniformly require *se* (or another overt reciprocal element) in sentences that express reciprocity.

7 Our characterization does not extend to ‘object-oriented’ reciprocals (Knjazev 2007): verbs that express reciprocity in the theme of a predicate, which may be expressed as an object of a transitive VP, as in (ia), or as the subject of the corresponding *se* form (ib). Notably, in constructions where *se* can be omitted, this ‘theme-oriented’ reciprocal reading may also be expressed without *se*, as exemplified by the analytic causative in (ii). However, since these verbs do not exhibit the same agent/patient alternation as the predicates in (29), we do not include them in our characterization.

8 *Se*-clauses with lexical reciprocal verbs are consistently ambiguous between the interpretations associated to the lexical or the grammatical reciprocal strategies, primed respectively in (33) and (34) by contextual information. However, we will see that this ambiguity can be systematically resolved: environments without *se* are unambiguity associated to the lexical strategy (§4.3), whereas overt adverbials are only consistent with grammatical reciprocity (§6.1).

9 With two participants, the natural reading of grammatical reciprocals is strong reciprocity, which requires two unidirectional relations. As we see below, with lexical reciprocals, weaker reciprocal readings that allow one unidirectional relation are also possible with some verbs. We do believe that weak reciprocity is also easier with lexical reciprocals when the subject refers to a larger number of participants, but this question requires further study.

10 A possible exceptional case is *the bunk beds are on top of one another* (Dalrymple et al. 1998), but see important empirical caveats in Mari (2013).

11 The lack of symmetry requirements for lexical reciprocal predicates has been substantiated experimentally. Kruitwagen et al. (2022) demonstrate that for many Dutch speakers, lexical reciprocals may relax the requirement that both participants are active, as long as there is an intentional collective involvement of participants in the action.

12 Dimitriadis (2008b) refers to lexical reciprocal events as ‘irreducibly symmetric’. While we subscribe to the view that lexical reciprocal meanings are not uniformly reducible to the meaning of the corresponding transitive meaning, we do not adopt Dimitriadis’s assumption that such events are necessarily symmetric in involving the corresponding transitive in both directions.

13 With plain reciprocals, the semantic distinction between pseudo-reciprocity readings and the interpretations of grammatical reciprocals is not easy to observe. There is hardly any semantic difference between forms such as *x and y meet* and *x and y meet each other*.

14 Note that in the case of the verb ‘kiss’, the pseudo-reciprocal reading may entail a plain reciprocal reading: if two individuals are involved in a mutual kiss, it follows that each might have been kissing the other. However, this is a property of the predicate ‘kiss’, and not an entailment relation that holds systematically between the relations denoted by lexical and grammatical reciprocal entries. Notably, with a verb like ‘leave/break up’, a collective form does not entail two unidirectional relations.

15 Kruitwagen et al. (2022) show that this latter kind of scenario is supported by lexical reciprocal predicates in Dutch or English, as long as the two individuals involved share a collective intention towards the hug. We believe that the same judgment extends to the Romance languages under consideration.

16 In the case of the verb ‘hug’, the reading associated to the lexical reciprocal entry does not entail two unilateral relations of the corresponding transitive (e.g., *x and y hug* ⇏ *x hugs y and y hugs x*), nor is it entailed by two transitive relations (e.g., *x hugs y and y hugs x* ⇏ *x and y hug*). However, with the majority of lexical reciprocal verbs that we are aware of, the entailment relation holds in one of the two directions, which makes it harder to use the zeugma test. Thus, while the predictions of the ambiguity analysis are borne out with the predicate ‘hug’, the same test does not allow us to test the same question with other lexical reciprocal predicates due to factors that are independent of the ambiguity question.

17 This generalization holds for languages such as Greek, English, Dutch, and Hebrew, where lexical reciprocity is expressed by a (possibly empty) verbal morpheme, and grammatical reciprocity is associated with a pronominal strategy. However, the pattern does not extend to verbal derivational morphology, where the discontinuous reciprocal construction is available with any transitive verb bearing the reciprocal morpheme. Some examples include Swahili (ia), Japanese (ib), and Malagasy (ic):

18 In Table 2, where we summarized the constructions where the omission of *se* is allowed with lexical reciprocals, the classification ‘finite clauses’ encompasses the discontinuous reciprocal construction. We know of only a few cases where acceptability differs between *se* omission in discontinuous constructions and simple finite clauses with a plural subject. For example, with the BP verb *consultar* ‘consult’, the omission of *se* in finite clauses with a plural subject seems hard for native speakers (ia), yet this verb allows discontinuous reciprocity (ib) without *se*, and has other characteristics of lexical reciprocals (see Appendices). Accordingly, *consultar* supports our identification of verbs that can express reciprocity without *se* with the Romance lexical reciprocals (Table 2).

19 In British English, which often allows plural agreement for singular group NPs, grammatical reciprocity is acceptable, though as in other varieties of English, this is not possible with singular agreement (de Vries 2015):

In Hungarian, by contrast, reciprocal anaphors are licensed by singular verbs (ii):

20 Unlike English *shave*, the verb *depilar* (BP) or *depilare* (It) refers to the removal of body hair, without any specification of the instrument used. Yet, we use *shave* as a translation, due to the similar semantic effects of pseudo-reflexivity discussed below.

21 Volition in such *n*-ary constructions, or the lack thereof, only arises by virtue of the meaning of the *n*-ary predicate. Thus, *Ali shaved himself* does not require Ali’s volition for the same reason that *Ali shaved John* does not: both sentences can be true if someone forced Ali to do the actions.

22 As for the omission of *se* with lexical reciprocals (Sect. 3.1), we expect possible variation among BP speakers on the acceptability of grammatical reciprocity without *se*. However, we generalize that if a speaker accepts the omission of *se* with *um o outro* and verbs that are cross-linguistically transitive-only (e.g., ‘describe’ or ‘thank’), then the speaker accepts the omission of *se* with *um o outro* for all transitives.

23 The observations presented in this section do not apply to the Italian element *se stesso* ‘himself’, which is incompatible with *se* (i):

24 Labelle (2008) does not mention example (82). The contrast that she provides between example (83) and ordinary transitive verbs is not as minimal as the contrast in (82) and (83), though this does not affect her main argument.

25 The only denotation Labelle proposes for Active Voice appears on the meaning she adopts from Kratzer (Labelle 2008, p.836). However, the denotation for Active Voice in (Labelle 2008, (8b)) is only defined for one-place predicates over events, and not for two-place predicates like Labelle’s analysis of *autoanalyzer*. Thus, Labelle’s account of the impossibility of Active Voice with *autoanalyzer* (Labelle 2008, pp.844–5) is not based on her other assumptions.

26 Labelle (2008) combines Kratzer’s operator of Event Identification and her own analysis of *se*. We pack Event Identification into the meaning of Active Voice. This creates a more uniform type strategy but with the same analysis by Kratzer.

27 In Sect. 4.3 we argued in favor of ambiguity between lexical reciprocal and grammatical reciprocal entries. It should be noted that in this paper we are not taking a firm commitment with respect to the ambiguity between grammatical reflexivity and grammatical reciprocity. For concreteness, we assume ambiguity between covert reflexive and reciprocal operators, but this can be replaced by a theory of underspecified meanings as in Murray (2008), Cable (2012), Haug and Dalrymple (2018). However, more recent results in Palmieri (2020) indicate that the question of ambiguity vs. underspecification in Romance between reciprocal and reflexive operator is more complex, hence the proposal here is not necessarily contradicted by the arguments in Cable (2012).

28 As we mentioned in Sect. 2, *se* can also be associated with middle interpretations that are not reflexive or reciprocal. We do not consider such cases as R-marked. While our analysis of *se* has the potential of being extended to other cases where no Agent variable is introduced (such as anticausatives), this goes beyond the scope of the current paper. For now, we follow Labelle (2008) in treating the reflexive/reciprocal *se* separately from the middle/anticausative *se*.

29 An anonymous NLLT reviewer points out that since the pseudo-reflexive interpretation is logically more general than plain reflexivity, we might postulate an unambiguously pseudo-reflexive reading for such sentences. However, for reasons of theoretical parsimony, we analyze lexical reflexives without *se* using the same ambiguity analysis that we adopted for lexical reciprocals, which was argued for in Sect. 4.3, in relation to example (44).
